# Clioquinol as a new therapy in epilepsy: From preclinical evidence to a proof‐of‐concept clinical study

**DOI:** 10.1111/epi.18536

**Published:** 2025-07-05

**Authors:** Karin Thevissen, Annelii Ny, Daniëlle Copmans, Jana Tits, Kenichi Kamata, Eva Gielis, Kevin Longin, Jo Sourbron, Peravina Thergarajan, Tracie Huey‐Lin Tan, Idrish Ali, Nigel C. Jones, Terence J. O'Brien, Mastura Monif, Bridgette D. Semple, Christine Germeys, Benedetta Frizzi, Virginia Minniti, Sebastian Perrone, Carole L. Linster, Ilaria Elia, Ludo Van Den Bosch, Bruno P. A. Cammue, Arnout Voet, Lieven Lagae, Peter de Witte

**Affiliations:** ^1^ Centre of Microbial and Plant Genetics, Department of Microbial and Molecular Systems KU Leuven Leuven Belgium; ^2^ Department of Microbiology, Immunology, and Transplantation KU Leuven Leuven Belgium; ^3^ Laboratory for Molecular Biodiscovery, Department of Pharmaceutical and Pharmacological Sciences KU Leuven Leuven Belgium; ^4^ Laboratory for Biomolecular Modeling and Design, Department of Chemistry KU Leuven Leuven Belgium; ^5^ Department of Development and Regeneration, Section of Pediatric Neurology University Hospitals Leuven Leuven Belgium; ^6^ Member of European Reference Network EpiCARE; ^7^ Center for Medical Genetics Ghent University Hospital Ghent Belgium; ^8^ Department of Neuroscience, School of Translational Medicine Monash University Melbourne Victoria Australia; ^9^ Department of Medicine Royal Melbourne Hospital, University of Melbourne Parkville Victoria Australia; ^10^ Department of Neurology Alfred Health Melbourne Victoria Australia; ^11^ Department of Neurology Royal Melbourne Hospital Melbourne Victoria Australia; ^12^ Department of Neurosciences and Leuven Brain Institute KU Leuven Leuven Belgium; ^13^ Vlaams Instituut voor Biotechnologie (VIB), Center for Brain & Disease Research Leuven Belgium; ^14^ Department of Cellular and Molecular Medicine KU Leuven Leuven Belgium; ^15^ Luxembourg Centre for Systems Biomedicine, Enzymology & Metabolism group University of Luxembourg Belvaux Luxembourg

**Keywords:** de novo glycine biosynthesis, mode of action, therapeutic intervention, translational neuroscience

## Abstract

**Objective:**

Drug‐resistant epilepsy (DRE) affects >25 million people worldwide and is often associated with neuroinflammation. Increasing evidence links deficiency or malfunctioning of the enzyme phosphoglycerate dehydrogenase (PHGDH), which converts 3‐phosphoglycerate to generate serine and the neurotransmitter glycine, with (drug‐resistant) epilepsy. Moreover, PHGDH, which is primarily expressed in astrocytes within the brain, has been identified as a critical enzyme in driving macrophage polarization toward an anti‐inflammatory state. Hence, PHGDH activators may be beneficial for treating DRE by exhibiting both antiseizure and anti‐inflammatory activity. The objective of this study was to identify such PHGDH activators.

**Methods:**

We screened a drug repurposing library for PHGDH activators and assessed their antiseizure and anti‐inflammatory properties using various zebrafish and mouse epilepsy models and explored the mechanistic consequences of activating PHGDH in a cell line, in astrocytes, and in zebrafish heads. Finally, we assessed the efficacy of clioquinol as add‐on treatment in three severe DRE patients in a clinical open pilot proof‐of‐concept study.

**Results:**

We identified haloquinolines from a drug repurposing library as potent activators of PHGDH. The most promising haloquinoline clioquinol can increase the catalytic activity of PHGDH up to 2.5‐fold, thereby increasing de novo glycine biosynthesis and resulting in reduced glutamate levels. Moreover, we show that clioquinol has PHGDH‐dependent antiseizure activity as well as anti‐inflammatory properties in vivo using various zebrafish and mouse epilepsy models. Finally, we demonstrate the efficacy of clioquinol as add‐on treatment in severe DRE patients; two patients showed a 37%–47% reduction in seizure frequency, and all three patients noted a positive impact on quality of life and seizure severity.

**Significance:**

Increasing activity of PHGDH is a promising new approach to treat DRE.

**Key points**

- Clioquinol can activate the enzyme PHGDH.
- Clioquinol reduces levels of the excitatory neurotransmitter glutamate.
- Activating PHGDH via clioquinol can reduce seizures and inflammation.
- Patients suffering from DRE show improved outcomes upon treatment with clioquinol.

## 1 INTRODUCTION

Epilepsy is a prevalent neurological disorder affecting >80 million individuals worldwide,^1^ often accompanied by neuroinflammation that can both cause and result from epileptic activity.^2^ Antiseizure medications (ASMs) are commonly used to control seizures, whereas anti‐inflammatory treatment is used in certain epilepsy syndromes, such as infantile spasms.^3^ However, approximately one third of patients are classified as having drug‐resistant epilepsy (DRE), as they do not adequately respond to current ASMs despite the availability of more than 25 drugs on the market.^4^

**FIGURE 1:**
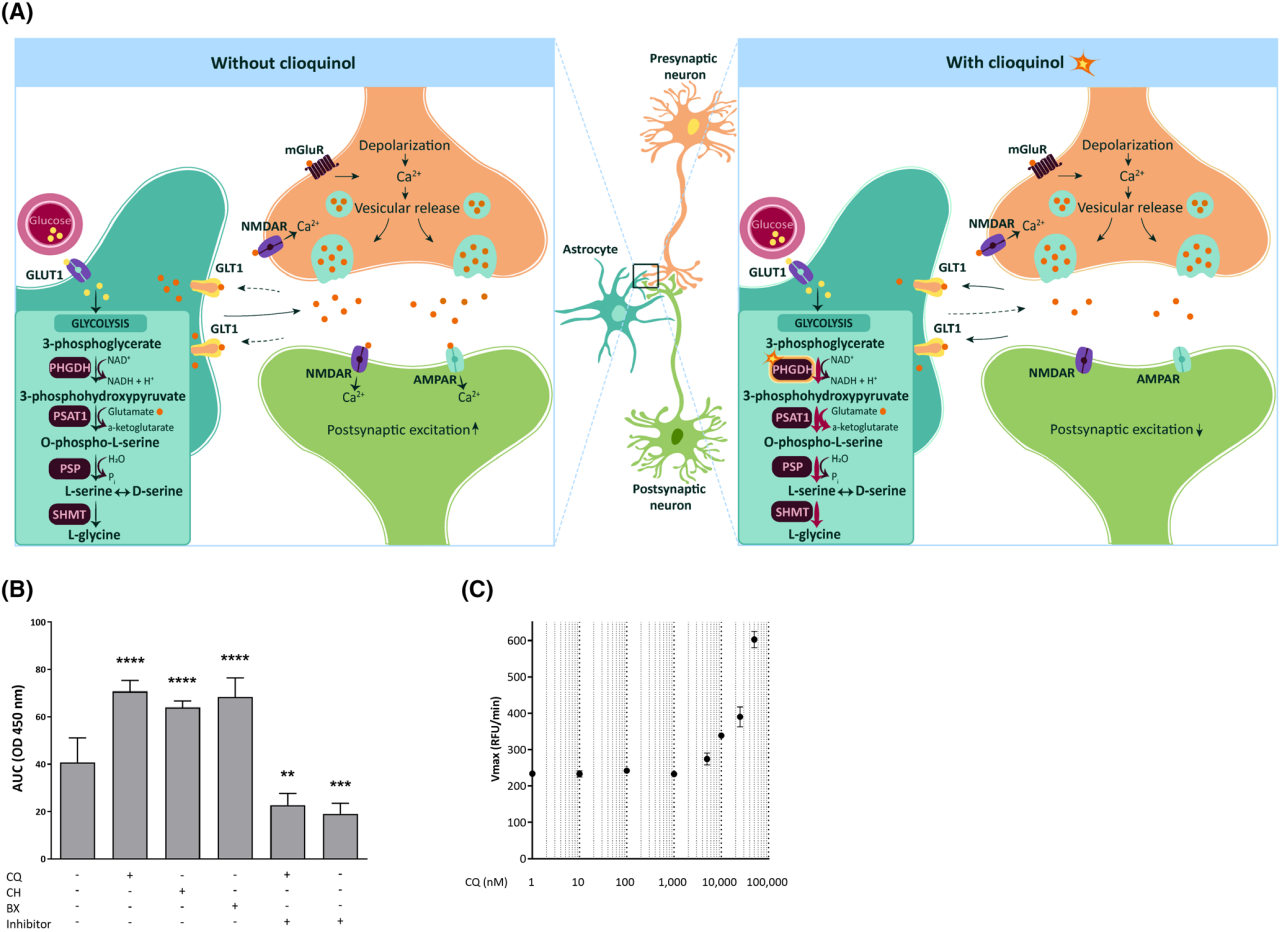
Phosphoglycerate dehydrogenase (PHGDH) activation by haloquinolines. (A) Schematic representation of de novo glycine/serine biosynthesis. Clioquinol (CQ) activation of PHGDH results in increased 3‐phosphoglycerate to 3‐phosphonooxypyruvate and downstream glycine synthesis, involving phosphoserine aminotransferase 1 (PSAT1)'s conversion of glutamate to α‐ketoglutarate. (B) PHGDH activity with or without 25 μmol·L^−1^ chloroxine (CH), 25 μmol·L^−1^ broxyquinoline (BX), or 25 μmol·L^−1^ CQ, alone and for CQ also in combination with 10 μmol·L^−1^ of the PHGDH inhibitor CBR‐5884. PHGDH activity was measured during 1 h with optical density (OD) = 450 nm as readout in a colorimetric assay, representing nicotinamide adenine dinucleotide (NADH) generated. Data are shown as mean area under the curve (AUC) ± SD in at least three biological repeats. Data shown are mean ± SD (*n* ≥ 5 in two biological repeats). Statistical differences: **p < .01, ***p < .001, ****p < .0001 by one‐way analysis of variance with Dunnett multiple comparisons test. (C) *V*_max_ (*K*_cat_) of serine PHGDH in the presence of different concentrations of CQ. *V*_max_ was calculated based on colorimetric change due to NADH‐induced resazurin to resorufin conversion at increasing (.5–5 mmol·L^−1^) 3‐phosphoglycerate and 100‐fold excess nicotinamide adenine dinucleotide. Data are mean ± SD of three biological repeats. AMPAR, α‐amino‐3‐hydroxy‐5‐methyl‐4‐isoxazolepropionic acid receptor; GLT1, glutamate transporter 1; GLUT1, glucose transporter 1; mGluR, metabotropic glutamatergic receptor; NMDAR, N‐methyl‐D‐aspartate receptor; PSP, phosphoserine phosphatase; SHMT, serine hydroxymethyltransferase.

This study explores phosphoglycerate dehydrogenase (PHGDH) as a potential novel therapeutic target for the treatment of DRE. Mounting evidence indicates a link between seizures and dysfunction of PHGDH, either due to genetic mutations in the *PHGDH* gene^5^, ^6^ or through PHGDH inhibition.^7^, ^8^ Additionally, PHGDH has been identified as a critical enzyme in driving macrophage polarization toward an anti‐inflammatory state.^9^ PHGDH catalyzes the conversion of the glycolytic intermediate 3‐phosphoglycerate to 3‐phosphonooxypyruvate (3‐PHP), which is the rate‐limiting step in de novo serine/glycine biosynthesis.^10^ Subsequently, 3‐PHP is converted into O‐phospho‐L‐serine by phosphoserine aminotransferase 1 (PSAT1) in a glutamate‐linked transamination reaction, and finally into L‐serine by phosphoserine phosphatase. Ultimately, a racemase and serine hydroxymethyltransferase convert L‐serine into D‐serine and L‐glycine, respectively (Figure 1A).

PHGDH is primarily expressed in astrocytes within the brain,^11^, ^12^, ^13^, ^14^ underscoring their central role in supplying L‐serine and other metabolites to other neural cell types. Astrocytes are a type of glia cell that are essential for various metabolic, structural, homeostatic, and neuroprotective brain functions.^15^ Astrocyte‐specific targets involved in pathological mechanisms are currently being investigated as potential avenues for novel therapies, including approaches like genetic reprogramming.^16^, ^17^ We hypothesized that activators of PHGDH may represent a fundamentally new therapeutic strategy, potentially combining antiseizure and anti‐inflammatory activity in an unique astrocyte‐specific manner. The metabolite 2‐phosphoglutarate was previously shown to activate PHGDH,^18^ indicating that the concept of screening for novel PHGDH activator drugs is feasible.

Here, we identified several haloquinolines as PHGDH activators, with clioquinol (CQ), an antifungal and antiprotozoal drug,^19^ showing the most promising results. We assessed the antiseizure activity of CQ in zebrafish epilepsy models and the mouse 6‐Hz focal seizure model, and its anti‐inflammatory properties in the self‐sustained status epilepticus (SSSE) mouse epilepsy model. Additionally, the mechanistic consequences of activating PHGDH in a PHGDH‐dependent cell line and in human astrocytes as well as in zebrafish heads were explored. Finally, we assessed the efficacy of CQ as add‐on treatment in severe DRE patients in a clinical open pilot proof‐of‐concept study.

## 2 MATERIALS AND METHODS

### 2.1 Compound preparations

CQ, broxyquinoline, and CBR‐5884 were purchased from Sigma‐Aldrich and chloroxine from TCI Europe; compound stock solutions were prepared in dimethylsulfoxide (DMSO; VWR International). Zinc sulphate heptahydrate, copper (II) sulphate pentahydrate, and L‐serine were from Sigma‐Aldrich; stock solutions/compounds were prepared in milliQ water in .3× Danieau solution (1.5 mmol·L^−1^ hydroxyethylpiperazine ethane sulfonic acid [HEPES], pH 7.6, 17.4 mmol·L^−1^ NaCl, .21 mmol·L^−1^ KCl, .12 mmol·L^−1^ MgSO_4_, and .18 mmol·L^−1^ Ca[NO_3_]_2_). Fenfluramine was from Prof. Ceulemans of Child Neurology, University Hospital Antwerp, Belgium. Ethyl ketopentenoate (EKP) was synthesized by Molecular Design and Synthesis (Prof. Wim De Borggraeve, KU Leuven, Belgium) as described.^20^

### 2.2 PHGDH enzymatic activity assay

PHGDH enzymatic activity was measured using human PHGDH (BPS Bioscience) in combination with a colorimetric PHGDH activity assay kit (Abcam) as described.^21^ Prior to the assay, compound stock solutions were diluted in PHGDH assay buffer to obtain a 5% DMSO solution, and human PHGDH enzyme was diluted in assay buffer at a concentration of .38 mg/mL. DMSO background was .5%. Absorbance at 450 nm (optical density [OD] = 450 nm) over time was measured as a readout for the amount of nicotinamide adenine dinucleotide (NADH) generated through PHGDH activity. The area under the curve (OD = 450 nm plotted against time [min]) represents PHGDH activity during 1 h. One‐way analysis of variance (ANOVA) with Dunnett multiple comparisons test was performed to assign significant differences compared to the control condition (.5% DMSO).

### 2.3 Enzyme kinetics assessment of recombinant serine PHGDH

*Escherichia coli* BL21 (DE3) cells were transformed with serine phosphoglycerate dehydrogenase (sPHGDH; N‐terminal domains of PHGDH) encoded in pET28.^22^ After induction of protein expression by .5 mmol·L^−1^ isopropyl thiogalactoside, cells were harvested and suspended in 100 mmol·L^−1^ Tris–HCl, pH 8.0, 150 mmol·L^−1^ NaCl, 1 mmol·L^−1^ dithiothreitol, .1 mmol·L^−1^ phenylmethylsulfonyl fluoride, and sonicated on ice. The supernatant of the lysate was loaded onto a 5‐mL nickel‐Sepharose column (Qiagen) and the nickel resin eluted with 50 mmol·L^−1^ Tris–HCl, pH 8.0, 150 mmol·L^−1^ NaCl, 500 mmol·L^−1^ imidazole. The eluted fractions were concentrated to 3 mL using Amicon centrifugal filter units (Millipore) and loaded onto a size‐exclusion column (GE Healthcare) with 50 mmol·L^−1^ HEPES, pH 7.5, 100 mmol·L^−1^ NaCl, 1 mmol·L^−1^ EDTA.

Kinetic assays were performed in 96‐well transparent film bottom plates (Greiner) at 37°C. The reaction solution was 50 mmol·L^−1^ HEPES, pH 7.5, 100 mmol·L^−1^ NaCl, 1 mmol·L^−1^ EDTA, 1 mmol·L^−1^ nicotinamide adenine dinucleotide [NAD^+^], .5–5 mmol·L^−1^ 3‐phosphoglycerate (3‐PG), .1 mmol·L^−1^ resazurin, 7.5% DMSO, 50 μmol·L^−1^ sPHGDH, and 0–50 μmol·L^−1^ CQ, and the reaction volume was 200 μL. CQ was not soluble under reaction conditions at higher concentrations. The enzymatic reaction was measured by detecting resorufin produced by reduction of resazurin by produced NADH. The fluorescence of resorufin (Ex: 550 nm/Ex: 580 nm) was measured by the plate reader (TECAN Safire). The initial rate was measured in three independent experiments and calculated by averaging. *V*_max_ values were calculated by Michaelis–Menten curve fitting using R.

### 2.4 Determination of serine, glutamate, and glycine in 4T1 breast cancer cell line and glucose tracing

The tumor‐derived 4T1 breast cancer cell line was provided by the Fendt Lab (VIB‐KU Leuven). 4T1 cells were plated in RPMI 1640 (Gibco 11875093) with 10% fetal bovine serum (FBS; Biowest S1400‐500), 1% sodium pyruvate 100 mmol·L^−1^ (Gibco 11360070), 1% HEPES 1 mol·L^−1^ (Gibco 15630080), and .1% 2‐mercaptoethanol (Sigma M3148) medium (complete RPMI medium). RPMI 1640 medium deprived in glucose, L‐glycine, L‐glutamine, and L‐serine (Cell Culture Technologies 1270RPM‐0324) was prepared according to the manufacturer protocol using sodium hydrogen carbonate (Sigma S5761). To this media, ^12^C glucose (2000 mg/L; Sigma G7021), ^13^C_6_ glucose (2000 mg/L; Sigma 389374), L‐glutamine (300 mg/L; Sigma G8540), 1% sodium pyruvate 100 mmol·L^−1^, 1% HEPES 1 mol·L^−1^, .1% 2‐mercaptoethanol, and 10% dialyzed FBS (dFBS), which has been processed using the Slide‐A‐Lyzer (Thermo Scientific 66130, 66493), were added, generating serine‐ and glycine‐deprived RPMI 1640 culture medium (Special RPMI 1640 medium). 4T1 cells (45.000/well) were plated in six‐well plates (CELLSTAR 657160) in 2 mL/well of complete RPMI 1640 medium. The day after, media were replaced with the RPMI 1640 depleted in serine and glycine with or without CQ. DMSO only was used as a control condition. After 24 h, media were aspirated, wells were washed with Dulbecco phosphate‐buffered saline, and the cells were quenched by contact with liquid nitrogen. Metabolite extraction and measurement using gas chromatography–mass spectrometry was performed as described.^23^, ^24^ Kruskal–Wallis test followed by Dunn multiple comparisons test (serine and glycine) and one‐way ANOVA with Dunnett multiple comparisons test (glutamate) were performed to assign significant differences compared to the control conditions (without CQ).

### 2.5 Zebrafish EKP seizure model and Dravet syndrome epilepsy model

Adult zebrafish (*Danio rerio*) of AB strain (Zebrafish International Resource Center) and *scn1Lab* strain (Dravet syndrome, gifted by Dr. Herwig Baier, Max Planck Institute of Neurobiology, Germany) were kept at 28°C, pH 6.5–7.8 and in a 14/10‐h light/dark regimen. Upon natural spawning, fertilized eggs were raised in embryo medium (.3× Danieau solution) at 28°C in continuous light until 7 days postfertilization (dpf). Zebrafish were raised and housed under identical conditions and in groups with a group size of 50–200 zebrafish larvae. Zebrafish larvae were randomly allocated to control and treatment groups. Different treatment groups were simultaneously measured per replicate experiment and treatment groups were alternated within the plate between experiments. Experiments were approved by the ethics committee of the University of Leuven (023/2017 and 027/2019) and the Belgian Federal Department of Public Health, Food Safety, and Environment (LA1210261).

The maximum tolerated concentration (MTC) of the compounds diluted in vehicle (VHC; embryo medium, 1% DMSO) for the zebrafish larvae was determined as described.^25^ To determine the MTC of CQ in combination with the PHGDH inhibitor CBR‐5884, larvae were treated with the MTC/2 concentration of CQ and a concentration range of CBR‐5884. To assess antiseizure activity of the compounds, AB and *scn1Lab* larvae were placed in a 96‐well plate (1 larva/well) and treated with 100 μL VHC or compound for 2 h at 28°C in the dark. For the EKP assay, 100 μL VHC or EKP (600 μmol·L^−1^ in VHC) was added to the wells, resulting in a final EKP concentration of 300 μmol·L^−1^. Locomotor activity was measured in a ZebraBox (Viewpoint) and expressed in “actinteg,” that is, the sum of all image pixel changes detected during the time window.^20^ Data were plotted as movement (mean actinteg units/5 min relative to EKP‐only treatment) during the 30‐min recording interval. For the Dravet syndrome assay, locomotor activity was expressed in “lardist,” that is, total distance covered by the larvae in large movements. Data were plotted as movement (mean lardist relative to the *scn1Lab* ^*−/−*^ larvae) during the last 10 min of a 40‐min recording period (first 30 min equals habituation, hence 2.5‐h treatment period prior to recording). Kruskal–Wallis test followed by Dunn multiple comparisons test was performed to assign significant differences compared to the control conditions.

Noninvasive local field potential (LFP) recordings of the optic tectum (midbrain) of 7 dpf zebrafish larvae^20^, ^25^ were performed to measure epileptiform brain activities. Larvae were treated with the compounds for 2 h (see above). For the EKP assay, 100 μL VHC or 600 μmol·L^−1^ EKP (300 μmol·L^−1^ working concentration) was added, followed by 12 min of incubation in the dark before assessment. Larvae were immobilized in 2% low‐melting‐point agarose (Invitrogen) by positioning of a single glass electrode containing artificial cerebrospinal fluid (124 mmol·L^−1^ NaCl, 2 mmol·L^−1^ KCl, 2 mmol·L^−1^ MgSO_4_, 2 mmol·L^−1^ CaCl_2_, 1.25 mmol·L^−1^ KH_2_PO_4_, 26 mmol·L^−1^ NaHCO_3_, and 10 mmol·L^−1^ glucose) above the optic tectum on the skin. LFP recordings were performed for 10 min. Data were analyzed using Clampfit 10.2 software (Molecular Devices Corporation) and MATLAB R2018 software for visual inspection, and by power spectral density (PSD) analysis, respectively.^25^ The PSD results were normalized against the VHC control, and the mean PSD per larva over a 10–90‐Hz frequency range was calculated per condition. Kruskal–Wallis test followed by Dunn multiple comparisons test was performed to assign significant differences compared to the control conditions.

### 2.6 Determination of glutamate, serine, and glycine levels in zebrafish heads

Seven‐dpf larvae (12/condition) were treated with or without 1 μmol·L^−1^ CQ in 1 mL of VHC (embryo medium, 1% DMSO) for 2 h at 28°C in the dark, followed by the addition of 1 mL VHC or 1 mL EKP solution (600 μmol·L^−1^ in embryo medium, 1% DMSO). After 1 min or 6 min, respectively, larvae were washed in embryo medium, and heads were isolated using a scalpel. Ten heads/condition were homogenized in 120 μL of antioxidant medium (.27 mmol·L^−1^ Na_2_EDTA·2H_2_O, .1 mol·L^−1^ acetic acid, 3.3 mmol·L^−1^ L‐cystine, 12.5 μmol·L^−1^ ascorbic acid) by twisting and moving a microtube homogenizer up and down 50 times at 4°C. Following centrifugation (12 000 × *g*, 15 min, 4°C), the supernatant was kept at −80°C until analysis. Glutamate, serine, and glycine concentration was analyzed by liquid chromatography with tandem mass spectrometry (LC–MS/MS). Results are shown as normalized glutamate, serine, or glycine amounts for the different treatments. To assign significant differences for the EKP model, unpaired Student *t*‐tests or Mann–Whitney test was performed. For the Dravet syndrome model, one‐way ANOVA with Tukey multiple comparisons test was performed.

### 2.7 Mouse 6‐Hz (44 mA) focal seizure model

Male Naval Medical Research Institute (NMRI) mice (Charles River Laboratories) were housed (5/cage) and maintained as described^25^ until the experiment was conducted. Experiments were approved by the ethics committee of the University of Leuven (027/2017) and the Belgian Federal Department of Public Health, Food Safety, and Environment (LA1210261). Antiseizure activities of CQ and fenfluramine were assessed as described.^25^ Briefly, NMRI mice were divided into different treatment groups, and 500 μL (adjusted to the individual weight) of VHC (.5% sodium carboxymethylcellulose/Tween 80 in .9% NaCl for CQ experiments and DMSO/polyethylene glycol 200 [50:50] for fenfluramine experiments) or treatment (CQ or fenfluramine dissolved in VHC) was injected intraperitoneally. After a 1‐h treatment period, corneas were anesthetized with .5% lidocaine, and seizures were induced by corneal electrical stimulation (6 Hz, .2‐ms rectangular pulse width, 3‐s duration, 44 mA) using an ECT Unit 5780 (Ugo Basile). The duration of psychomotor seizures for different CQ doses was calculated upon blinded video analysis. Kruskal–Wallis test followed by Dunn multiple comparisons test was performed to assign significant differences compared to VHC.

### 2.8 SSSE mouse epilepsy model

Seven‐week‐old C57bl/6/j mice (Alfred Medical Research & Education animal facility) were used for the gene expression study after approval from the local ethics committee (E/2077/2021/M). SSSE induction was performed as described.^26^ Mice received either CQ (5 mg/kg ip twice daily) or VHC injections for 1 week, which was initiated on the next day after the SSSE. CQ was suspended in 5% DMSO and 20% Kolliphor RH40 in .01 mol·L^−1^ phosphate‐buffered saline. Hippocampi were dissected and mRNA was extracted using a Nucleospin RNA Plus kit (Machery‐Nagel), and cDNA synthesis was performed using the Omniscript RT Kit (Qiagen). The real‐time quantitative polymerase chain reaction was completed using high‐throughput gene expression analysis based on microfluidic dynamic arrays.^26^ The gene expression levels for each sample were normalized to the geometric mean of housekeeping genes including *GAPDH*, *ACTB*, *HPRT1*, and *PPIA*. A two‐way ANOVA with Šídák multiple comparison test was performed to assign significant differences between the VHC and CQ as well as between sham and SSSE animals.

### 2.9 CQ clinical open pilot proof‐of‐concept study

Major inclusion criteria for participation in the study (EUDRACT 2020‐004511‐27) included informed consent, a diagnosis of DRE as defined by the International League Against Epilepsy, and age = 12–18 years and a minimum of four convulsive seizures in a 4‐week baseline period with no clustering of the seizures in 1 week (adapted from Lagae et al.^27^). CQ was formulated and manufactured by ACE Pharmaceuticals as a 100‐mg/mL syrup. The study was an add‐on open label pilot phase II trial.

Primary readout was seizure frequency/2 weeks. Prior to the administration of CQ, seizure frequency/2 weeks was recorded for 2–4 weeks (baseline; Figure S1). CQ was administered orally twice daily at 1 mg/kg/day (low dose) for 2 weeks followed by 4 mg/kg/day (high dose) for 6 weeks. During the trial, the concomitant ASM was kept stable (maximum three ASMs). Secondary outcome measures included change of seizure severity, impact on daily life, and quality of life, using the standardized questionnaires: National Hospital Seizure Severity Scale (NHS3)^28^ and Personal Impact of Epilepsy Scale (PIES).^29^ During visits 2 and 4, CQ plasma levels were assayed using high‐performance liquid chromatography (BioNotus). Standard adverse event reporting was conducted. Clinical examination, including visual screening (questionnaire and clinical examination) and motor examination were done at all visits.

### 2.10 Statistical analysis

All statistical analyses were performed using GraphPad Prism 10.4.1 software, and data are presented as mean ± SD. Outliers were removed by means of the Robust Regression and Outlier Removal (ROUT method (*Q* = 1%)). All datasets were assessed using the Shapiro–Wilk normality test. Data passing the test were analyzed using one‐way or two‐way ANOVA with Dunnett, Tukey, or Šídák multiple comparison tests, or by unpaired Student *t*‐test. Data failing the normality test were analyzed using Kruskal–Wallis test followed by Dunn multiple comparisons test, or Mann–Whitney test. Results were regarded as statistically significant at *p* < .05.

## 3 RESULTS

### 3.1 Haloquinoline CQ increases catalytic activity of human PHGDH

A novel function of PHGDH in reducing reactive oxygen species (ROS) levels via increased glutathione synthesis was uncovered in 2020.^30^ We previously identified various pharmaceutical compounds from a drug repurposing library that increased survival of yeast under lethal ROS‐inducing stress.^31^ Tanshinone was one of the hits from this screening and is known to increase the expression of *PHGDH*,^32^ which is consistent with a role for PHGDH in the normalization of ROS levels. Hence, we assessed the potential activation of human PHGDH by all previously identified hits from the screening^31^ and found that the haloquinolines CQ, chloroxine, and broxyquinoline resulted in increased PHGDH activity in an enzyme assay using human recombinant PHGDH and an NADH‐based colorimetric readout (Figure 1B). CQ was the most promising hit for further analysis, as it has been used clinically for a long time.^19^ Coadministration of the PHGDH‐specific inhibitor CBR‐5884^33^ with CQ completely blocked the induced PHGDH activation (Figure 1B), pointing to the PHGDH specificity of CQ's readout in the assay. Next, we assessed the effect of CQ on human recombinant PHGDH in an enzymatic assay using conditions that mimic a cellular context, that is, in the presence of PSAT1, the enzyme downstream of PHGDH, and a deproteinized cellular extract obtained from an HAP1 cell culture. The reaction was followed by measuring NADH spectrophotometrically and showed increased PHGDH activity by CQ in these conditions as well (Figure S2).

Previous kinetic studies with human PHGDH indicated that a truncated PHGDH variant, obtained by removal of the two regulatory C‐terminal domains, yielded an enzyme (sPHGDH) with similar catalytic efficiency as full‐length protein.^22^ We performed further enzymatic studies with sPHGDH to limit the impact of allosteric regulation, and in the presence of EDTA to exclude effects due to metal chelation by CQ.^34^ Activation of sPHGDH by CQ was observed from the turnover of 3‐PG and NAD^+^ at CQ concentrations up to 50 μmol·L^−1^ (Figure S3). Further kinetic studies performed with varying 3‐PG concentration and surplus NAD^+^ demonstrated a concentration‐dependent increase of the maximal reaction rate *V*_max_ (from 239.4 ± 12.7 RFU/min to 611.7 ± 22.6 RFU/min; Figure 1C), implying a 2.5‐fold improvement of the catalytic constant *K*_cat_. As no plateau of *V*_max_ could be reached due to low solubility of CQ, the EC_50_ of CQ could not be determined. These results prove that CQ directly engages with PHGDH via the active site domains of PHGDH and increases its activity.

### 3.2 CQ enhances de novo glycine biosynthesis and results in decreased glutamate levels

**FIGURE 2:**
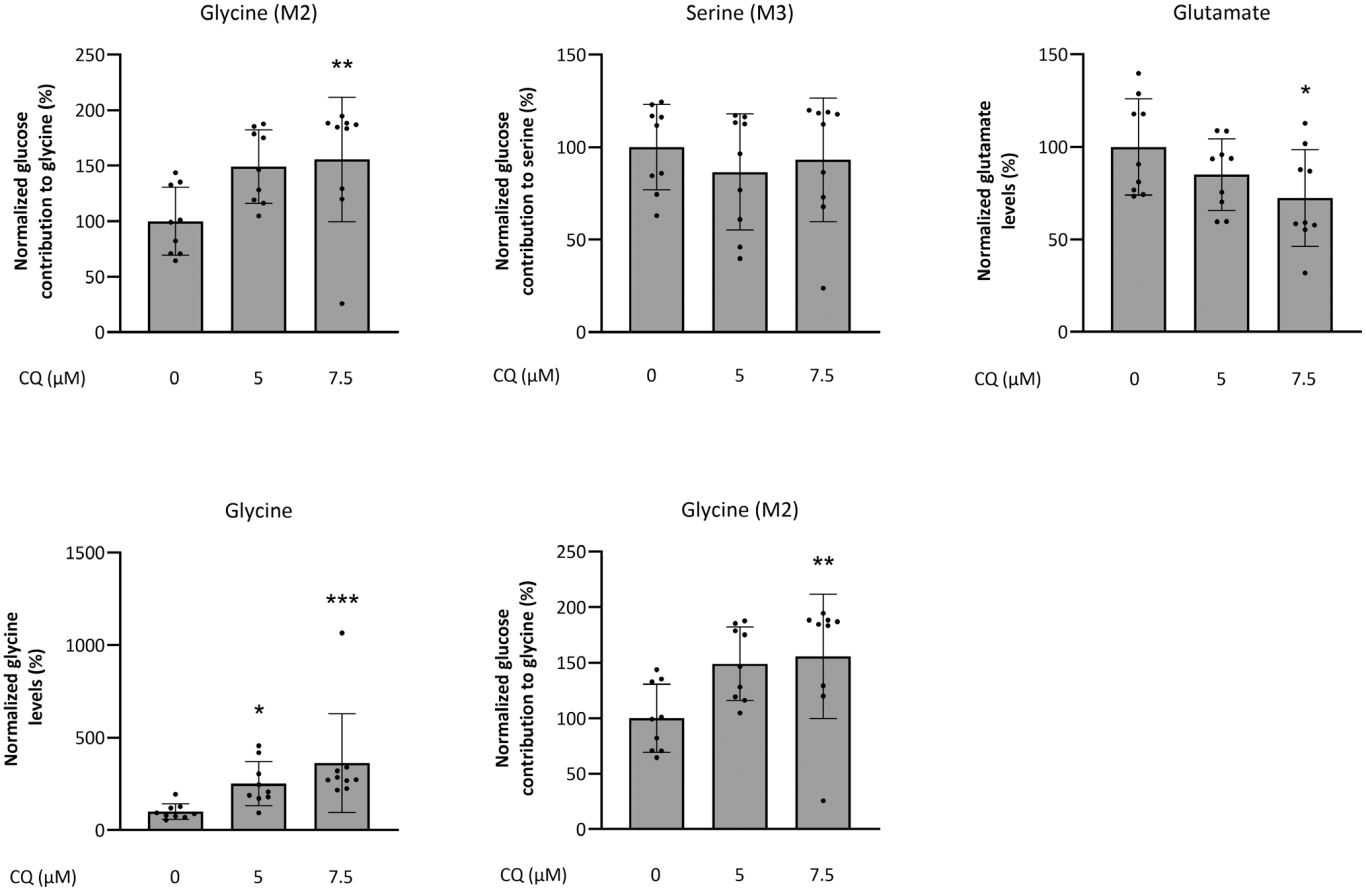
Impact of clioquinol (CQ) on the phosphoglycerate dehydrogenase‐driven de novo glycine biosynthesis on 4T1 breast cancer cells. 4T1 breast cancer cells were incubated with 0 μmol·L^−1^ (*n* = 9), 5 μmol·L^−1^ (*n* = 9), and 7.5 μmol·L^−1^ CQ (*n* = 9) in serine‐ and glycine‐free medium in three biological repeats. Intracellular levels of serine, glycine, and glutamate were measured using gas chromatography–mass spectrometry, and the fraction of glucose in serine and glycine upon ^13^C_6_ glucose tracing was calculated and normalized to vehicle (0 μmol·L^−1^) ± SD. M2 and M3 refer to the number of ^13^C‐labeled carbon atoms in glycine and serine, respectively. Statistical differences: *p < .05, **p < .01, ***p < .001 by Kruskal–Wallis test followed by Dunn multiple comparisons test (serine and glycine) and one‐way analysis of variance with Dunnett multiple comparisons test (glutamate).

By increasing the activity of PHGDH, we hypothesize that more 3‐PHP is produced, which in turn will be converted to more o‐phospho‐L‐serine by PSAT1 and ultimately to more serine and/or glycine. As PSAT1 utilizes glutamate,^35^ which is an excitatory neurotransmitter,^36^ PHGDH activation should also result in reduced levels of intracellular glutamate. Hence, we measured intracellular levels of serine, glycine, and glutamate and performed ^13^C_6_ glucose tracing in CQ‐treated and untreated 4T1 breast cancer cells, which have a high dependency on serine/glycine biosynthesis, in serine‐ and glycine‐free medium. CQ treatment of 4T1 cells combined with ^13^C_6_ glucose tracing revealed no difference in intracellular serine levels and glucose contribution to serine (Serine (M3)) compared to 1% DMSO control, excluding an increase of serine biosynthesis levels upon CQ treatment (Figure 2). However, the same treatment resulted in an increase of intracellular glycine levels, significant at 7.5 μmol·L^−1^ CQ, coupled with a higher glucose contribution to glycine (Glycine (M2)), significant at both 5 and 7.5 μmol·L^−1^ CQ. These findings suggest an increased glycine biosynthesis upon CQ treatment. Additionally, the significant decrease in glutamate intracellular levels observed in the 7.5 μmol·L^−1^ CQ condition confirms the increased usage of this metabolite by PSAT1 to ultimately support glycine metabolism.

Next, as PHGDH is mainly expressed in astrocytes in the brain, we subsequently assessed glycine and glutamate levels in human induced pluripotent stem cell‐derived astrocytes treated with CQ. In line with the breast cancer cell line data, we found that treatment of astrocytes with CQ resulted in significantly increased intracellular glycine levels compared to the VHC‐treated controls, providing cellular proof‐of‐concept of PHGDH activation by CQ. We also observed a trend toward reduced levels of intracellular glutamate (*p* = .4; Figure S4). Overall, these data indicate that CQ can affect the intracellular levels of two key neurotransmitters, glycine and glutamate, through the activation of PHGDH.

### 3.3 CQ shows antiseizure activity in chemical and genetic zebrafish seizure models

First, we confirmed the association between PHGDH dysfunction and seizures in zebrafish, using the PHGDH‐specific inhibitor CBR‐5884. We assessed the effect of CBR‐5884 at its MTC (1 μmol·L^−1^) on the brain activity of 7‐dpf zebrafish larvae via LFP recordings (Figure S5). In line, we observed a significant increase in epileptiform events in larvae treated with CBR‐5884 compared to VHC only‐treated larvae, thereby demonstrating that CBR‐5884 induces epileptiform brain activity in zebrafish wild‐type larvae.

**FIGURE 3:**
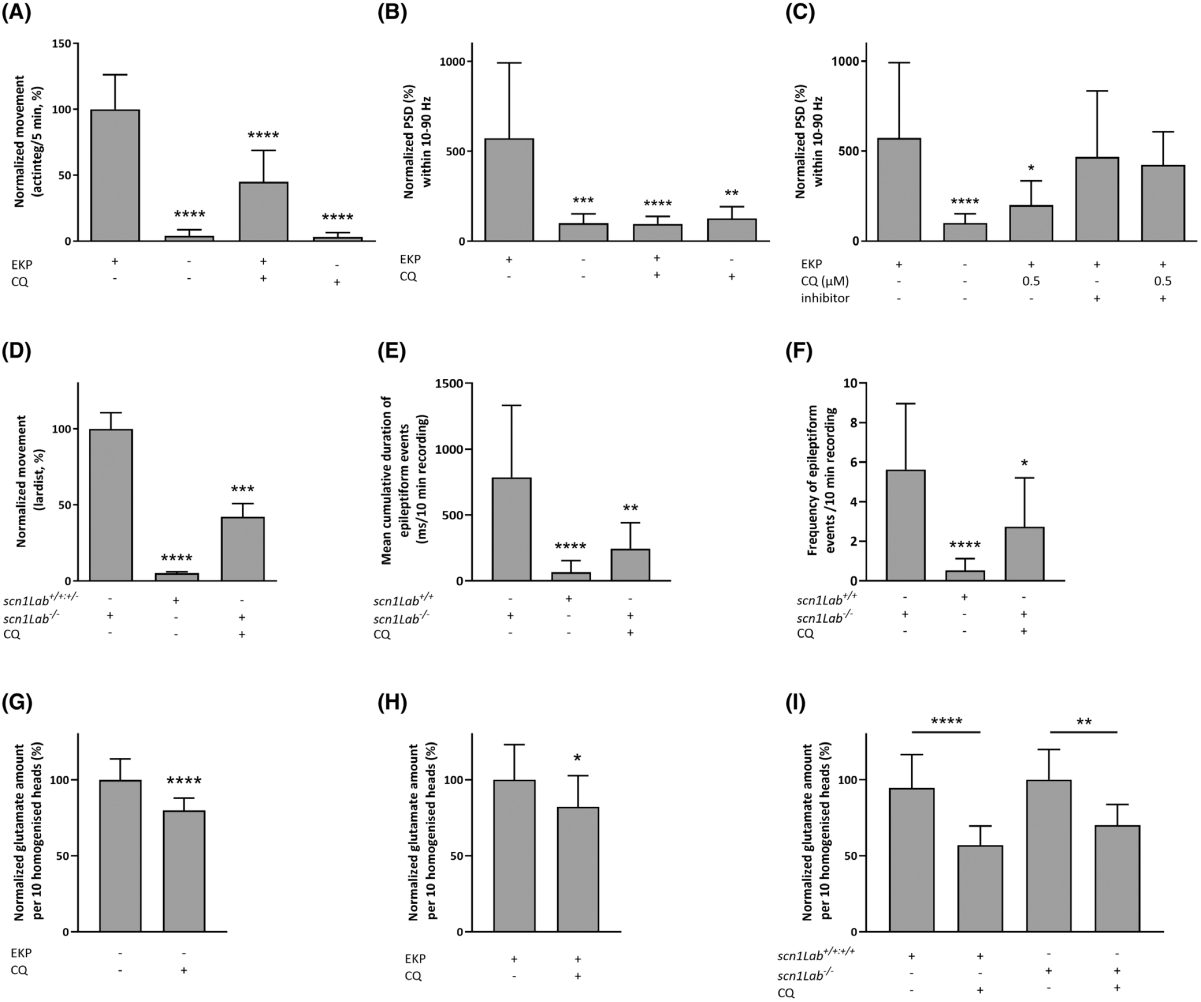
Antiseizure activity of clioquinol (CQ) in ethyl ketopentenoate (EKP) and Dravet syndrome (*scn1Lab* ^*−/*−^) zebrafish epilepsy models. (A, D) Locomotor activity of larvae in (A) response to EKP and (D) Dravet syndrome, with or without 1 μmol·L^−1^ CQ. (A, D) Activity expressed as (A) mean actinteg units per 5 min ± SD during 30 min, relative to EKP‐only treatment; or (D) normalized lardist per 100 s ± SD, relative to vehicle (VHC; 1% dimethylsulfoxide). (B, C) Electrophysiological activity of larvae expressed as mean normalized power spectral density (PSD) within a 10–90 Hz frequency range per larva ± SD, relative to VHC. (E, F) Mean cumulative duration of epileptiform events (ms/10 min recording; E) and frequency of epileptiform events/10 min recording ± SD (F), relative to *scn1Lab* ^*−/−*^. For each experiment, more than 10 larvae were used in at least three biological repeats. (G–I) Glutamate levels in heads of wild‐type (G) and EKP‐treated (H) larvae, and in Dravet syndrome zebrafish (I), with or without 1 μmol·L^−1^ CQ. Results are expressed as normalized glutamate or glycine amount per 10 homogenized heads ± SD. Statistical differences: *p < .05, **p < .01, ***p < .001, ****p < .0001 by Kruskal–Wallis test followed by Dunn multiple comparisons test (A–F), Student *t*‐test (G, H), and one‐way analysis of variance with Tukey multiple comparisons test (I).

Next, we used the pharmacologically validated zebrafish EKP chemical seizure model^20^ and the Dravet syndrome genetic epilepsy model^37^ to investigate the activity of CQ against drug‐resistant seizures. EKP is a potent inhibitor of glutamate decarboxylase (GAD), a key enzyme involved in the regulation of neural network excitability through the conversion of glutamate to γ‐aminobutyric acid. The zebrafish EKP model holds clinical significance, as reduced GAD activity is linked to various forms of intractable epilepsy.^20^ We assessed the effect of CQ (MTC of 1 μmol·L^−1^) on locomotor and epileptiform brain activity in the zebrafish EKP seizure model. In the absence of EKP, CQ did not affect locomotor or brain activity in zebrafish. However, in the presence of EKP, CQ significantly reduced EKP‐induced elevated locomotor activity (Figure 3A) as well as EKP‐induced epileptiform brain activity (Figures 3B and S6). In contrast to .5 μmol·L^−1^ CQ alone, coadministration of CQ and CBR‐5884 at the MTC for this combination (i.e., .64 μmol·L^−1^ CBR‐5884) did not modify epileptiform brain activity induced by EKP (Figure 3C), suggesting a PHGDH‐dependent antiseizure action of CQ. The level of epileptiform activity upon treatment with .64 μmol·L^−1^ CBR‐5884, followed by the addition of EKP, did not differ significantly from that of larvae treated with EKP alone. None of the compounds or combinations resulted in a significant increase or decrease in epileptiform brain activity in the absence of EKP compared to the VHC treatment (data not shown).

We further assessed the effect of CQ in Dravet syndrome (*scn1Lab* ^*−/*−^) zebrafish, a model of genetic epilepsy. CQ (1 μmol·L^−1^) significantly reduced locomotor activity (Figure 3D), as well as the duration (Figure 3E) and frequency (Figure 3F) of epileptiform brain activity, in spontaneously seizing *scn1Lab* ^*−/−*^ zebrafish, compared with VHC treatment (embryo medium, 1% DMSO). As controls, *scn1Lab* ^*+/+*^ (wild‐type) larvae or a mixture of wild‐type and *scn1Lab* ^*+/−*^ (heterozygous) larvae, which do not exhibit seizures or epileptiform activity when assessed, were used (Figure 3E,F).

Next, we assessed glutamate, glycine, and serine levels in zebrafish heads following CQ treatment of wild‐type, EKP‐treated, and spontaneously seizing Dravet syndrome (*scn1Lab* ^*−/−*^) zebrafish via LC–MS/MS. Glutamate levels were consistently and significantly reduced by CQ treatment of wild‐type, EKP‐treated, and Dravet syndrome zebrafish as compared to VHC controls (Figure 3G–I), whereas glycine and serine levels were unaffected (Figure S7). These findings show that CQ can reduce in vivo brain levels of glutamate, the most abundant excitatory neurotransmitter, which likely contributes to its antiseizure activity.

### 3.4 CQ reduces seizure duration and increases anti‐inflammatory markers in mouse epilepsy models

**FIGURE 4:**
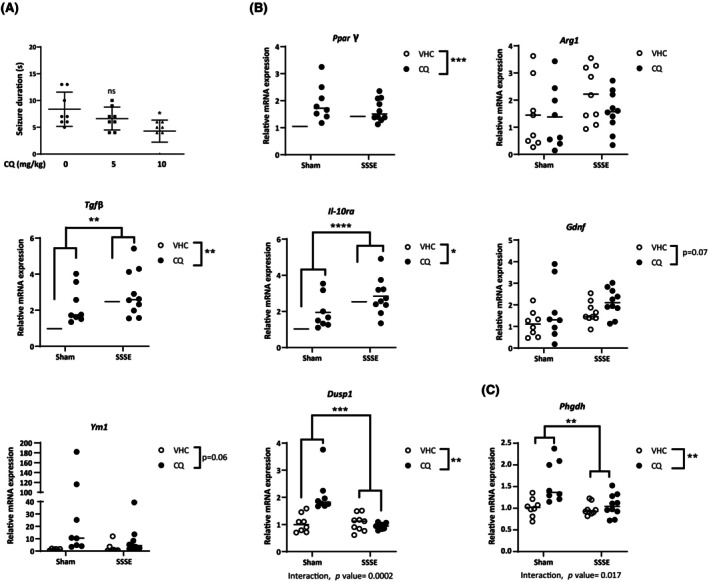
Antiseizure and anti‐inflammatory activity analysis of clioquinol (CQ) in the mouse 6‐Hz (44 mA) focal seizure and self‐sustained status epilepticus (SSSE) seizure models, respectively. (A) Drug‐resistant focal seizures were induced by electrical stimulation through the cornea, 60 min after intraperitoneal injection of vehicle (VHC; *n* = 8), CQ (5 mg/kg, *n* = 8), and CQ (10 mg/kg, *n* = 7). Mean seizure durations (±SD) are depicted. Statistical differences: *p < .05 by Kruskal–Wallis test followed by Dunn multiple comparisons test. (B, C) The hippocampal mRNA expression of anti‐inflammatory genes and phosphoglycerate dehydrogenase in CQ‐treated SHAM and SSSE animals is represented as dot plots with median represented by a line. Statistical differences: *p < .05, **p < .01, ***p < .001, ****p < .0001 by two‐way analysis of variance with Šídák multiple comparison test. ns, not significant.

The antiseizure activity of CQ was further evaluated in the mouse 6‐Hz focal seizure model. Seizures induced with 6‐Hz (44 mA) corneal stimulation are generally considered pharmacoresistant, as they are difficult to treat with currently available ASMs.^38^, ^39^ Our results show a significantly reduced seizure duration 60 min after intraperitoneal administration of 10 mg/kg CQ (*p* = .016) but not of 5 mg/kg CQ compared to VHC‐treated mice (Figure 4A). In addition, by comparing the CQ 5‐ and 10‐mg/kg conditions, a borderline significance (*p* = .07, Mann–Whitney) was observed.

As PHGDH has been identified as a critical enzyme in driving macrophage polarization toward an anti‐inflammatory state,^9^ we additionally investigated anti‐inflammatory activity of CQ in a mouse model of status epilepticus (SE).^26^ We assessed the expression of various anti‐inflammatory markers 1 week after induction of SE via electrical stimulation, comparing CQ treatment (5 mg/kg/day ip twice daily injections) initiated on the day following SSSE induction to the VHC treatment. At the end of the experiment, unilateral hippocampi were collected for gene expression analyses (Figure 4B,C). Two‐way ANOVA showed an overall effect of treatment, with CQ‐treated animals displaying a significant increase in the expression of *PHGDH*, as well as several anti‐inflammatory cytokines and neuroprotective genes such as *Tgfβ, Il‐10ra*, *Dusp1*, *PPAR*γ, *Gdnf* (*p* = .07), and *Ym1* (*p* = .06), when compared to VHC‐treated mice. TGFβ and IL10‐ra were also upregulated by SE compared to sham SE, whereas the level of DUSP1, which is involved in the resolution of inflammation, was significantly reduced by SE. The increase in the gene expression is somewhat less marked in the SE groups following the treatment, compared to sham groups; however, this was not supported, as no statistically significant interaction was observed between the two factors (SE vs. CQ treatment), and therefore a post hoc analysis to investigate this was not warranted. Several proinflammatory cytokines, such as Il1β, Il1α, Tnfα, Cd86, IL6, and Trem2, were significantly upregulated by SE, but no changes were induced by the CQ treatment except for *Il6* and *Trem2*, which showed a trend to increase (*p* = .06) in CQ compared to VHC treatment (Table S1). It is currently not clear how activation of PHGHD by CQ can result in increased expression of the *PHGDH* gene itself, and whether the observed CQ effects in vivo result from PHGDH activation, increased expression of PHGDH, or a combination of both. In conclusion, by activating PHGDH, we target drug‐resistant seizures as well as co‐occurring neuroinflammation.

### 3.5 CQ reduces seizures in adolescents suffering from DRE

To test the safety and potential efficacy of CQ in DRE patients, we designed an open pilot proof‐of‐concept (signal finding) study using CQ as an add‐on therapy. The study also included a follow‐up of pharmacokinetic parameters as well as the registration of potential adverse effects that might be linked to the development of subacute myelo‐optic neuropathy (SMON).^40^ Based on historical CQ pharmacokinetic studies in rodents,^41^, ^42^ we calculated the CQ target dose in man. Based on CQ serum levels upon single dose (oral) administration, a dose of 100 mg/kg po in mice is comparable with a dose of 7–9 mg/kg po in man.^41^ In hamsters, 50 mg/kg po results in 6.25‐fold lower CQ plasma levels as compared to the same CQ dose upon intraperitoneal administration. Hence, a target antiseizure CQ dose in mice of 10 mg/kg ip translates to an oral dose in rodents of 62.5 mg/kg or to a dose in man of approximately 4 mg/kg po. This target dose is much lower than doses associated with neurotoxicity/SMON (20–30 mg/kg/day)^43^ and fourfold lower than the CQ dose used for treatment of children with amoeba infections (15 mg/kg/day).^44^

In this study, three adolescents (CLIO‐002, CLIO‐003, and CLIO‐004; Table 1) with severe DRE who failed at least three ASMs were included. All three subjects fulfilled the criteria for Lennox–Gastaut syndrome. During the 4‐week baseline period, a minimum of four countable convulsive seizures was a requirement for inclusion. After a 2‐week low CQ dose exposure (1 mg/kg/day), patients received the target CQ dose (4 mg/kg/day) for 6 weeks. The primary outcome was the seizure frequency decrease during the higher CQ dose exposure compared to baseline. Secondary outcomes included seizure severity based on the NHS3^28^ and PIES^29^ as well as safety.

**TABLE 1:** Primary and secondary outcome parameters of the CQ proof‐of‐concept clinical study.

Outcome parameters	CQ dose, mg/kg/day	Patient		
		CLIO‐002	CLIO‐003^a^	CLIO‐004
Mean number of seizures per 2 weeks	0	5.0	12.7	13.3
	1	5.8	11.4	10.3
	4	5.1	8.0	7.0
	0	7.3	11.3	6.5
CQ plasma level, μg/mL [4 h postdose]	1	.508	<.25	<.25
	4	3.456	.965	.989
Seizure severity [NHS3 scale^b^]	0	12	16	6–10^c^
	1	8	10	6–10
	4	6	6	7–6
	0	5	12	10–8
Quality of life [PIES scale^d^]	0	50	65	41
	1	46	43	39
	4	19	46	38
	0	31	60	43

Abbreviations: CQ, clioquinol; NHS3, National Hospital Seizure Severity Scale; PIES, Personal Impact of Epilepsy Scale.

^a^ Dropped out after decision of parents because the effects on primary and secondary outcomes were not according to their expectations. Patient received the high CQ dose (4 mg/kg/day) for 3 weeks instead of 6.

^b^ NSH3 scale^25^ ranging from 1 (no serious seizures) to 26 (very serious seizures).

^c^ Three different types of seizures: tonic, tonic–clonic, drop attack.

^d^ PIES scale^26^ ranging from 0 (good well‐being) to 100 (bad well‐being).

In two of the three patients, a decrease in the seizure frequency upon receiving the target CQ dose relative to baseline was seen (37.0% and 47.4% in Patients CLIO‐003 and CLIO‐004, respectively). In view of the very refractory nature of their epilepsy, these results are promising and indicate a potential efficacy of CQ as add‐on treatment in refractory epilepsy. No adverse events, including ophthalmological or cognitive, related to the treatment were reported during the trial. The seizure severity measured using NHS3 also decreased in all three patients. A positive impact of the new treatment on comorbidities and quality of life using PIES was seen in all three patients, and was most pronounced in Patient CLIO‐002, although this patient had no formal decrease in seizure frequency. Our data showed very low serum concentrations at the low CQ dose exposure (≤.5 μg/mL) and stable levels at the higher dose (1–3.5 μg/mL), but still low compared to the CQ plasma level upon administration of a dose of 10 mg/kg/day in humans (7.6 μg/mL).^45^

## 4 DISCUSSION

Epileptic seizures can be treated by more than 25 marketed ASMs, which have different mechanisms of action (reviewed in Sills and Rogawski^46^). Nevertheless, one third of epilepsy patients suffer from DRE. In this study, we have identified compounds with anticonvulsant activity against drug‐resistant seizures by focusing on the enzyme PHGDH as a novel target. Increasing evidence links PHGDH deficiency with (drug‐resistant) epilepsy.^5^, ^6^, ^7^, ^8^ We identified various members of the haloquinoline drug class (e.g., CQ, chloroxine, and broxyquinoline) as PHGDH activators. Haloquinolines are halogenated 8‐hydroxyquinoline structures that have a wide variety of properties.^19^ CQ, which is an antifungal and an antiprotozoal drug, was selected and its anticonvulsant activity was assessed in the chemical (EKP‐based) seizure model and the genetic (Dravet syndrome) zebrafish epilepsy model.

Exploring the anticonvulsant mechanism of CQ, we found that CQ reduces glutamate levels under both in vitro and in vivo conditions. Notably, glutamate plays a critical role in excitatory neurotransmission, resulting in cellular and network hyperactivity, and it has been shown that when dysregulated, glutamatergic neurotransmission is fundamental to epileptogenesis and seizures.^47^ Because the activation of PHGDH also influences the synthesis of L‐serine, and consequently D‐serine and glycine, both of which serve as allosteric modulators at the glycine site of the N‐methyl‐D‐aspartate receptor,^48^ we also examined the concentrations of L‐serine and L‐glycine. In vitro, we observed an increase in L‐glycine but no change in L‐serine. In contrast, both compounds remained unchanged under in vivo conditions. The mechanistic details of this outcome are currently unclear, and further in‐depth investigations will be required to fully comprehend the downstream processing of L‐serine and glycine under these conditions.

We measured total glutamate levels in the zebrafish brain and found that CQ reduced glutamate concentrations by 15%–30%. Given that the intracellular glutamate concentrations (typically 1–2 mmol·L^−1^, primarily of neuronal and astrocytic origin) far exceed the extracellular glutamate fraction (1–3 μmol·L^−1^, but 10–20 μmol·L^−1^ under epileptogenic conditions)^49^ and that the extracellular space occupies a smaller part of brain tissue volume,^50^ we believe the observed reductions primarily reflect glutamate changes in the intracellular compartment. As we did not measure extracellular glutamate concentrations, the impact of this reduction on the steep concentration gradient between intracellular and extracellular compartments, an essential factor in evaluating seizure suppression and neuroprotection, remains unclear. However, previous studies have shown that oxcarbazepine and gabapentin, which similarly reduce total brain glutamate levels in zebrafish,^51^ can also suppress pharmacologically induced elevations in extracellular glutamate.^52^, ^53^ Therefore, it is anticipated that a reduction of 15%–30% in total glutamate content, as observed in the zebrafish brain in this study, could lead to significant decreases in extracellular glutamate surges under epileptic conditions.

Next, we further investigated the anticonvulsant activity of CQ in the mouse 6‐Hz (44 mA) focal seizure model, which is considered to mimic drug‐resistant seizures.^38^, ^39^ We observed a significant reduction in seizure duration upon treatment of mice with 10 mg/kg CQ as compared to VHC‐treated ones. The observed reduction in seizure duration by CQ treatment is comparable to that induced by fenfluramine (5 and 20 mg/kg) treatment in the 6‐Hz model (Figure S8). Fenfluramine was used as a benchmark because this prior antiobesity drug is currently being used as a novel ASM against DRE, including Dravet syndrome, Lennox–Gastaut syndrome, and Sunflower syndrome.^54^, ^55^ Additionally, we assessed CQ's anti‐inflammatory activity in a mouse epilepsy model of SSSE. We observed an overall effect of treatment with CQ that showed significant upregulation of the expression of some of the anti‐inflammatory genes, such as Tgfb, Il‐10ra, and PPARγ, which is demonstrative of an anti‐inflammatory effect of CQ. CQ‐mediated increase in the gene expression is somewhat less marked in the SE groups, compared to sham groups; however, this was not statistically supported, with no statistical interaction being observed between the two factors (SE vs. CQ treatment). It is also interesting that expression of proinflammatory genes that were increased by the SSSE were not altered by the CQ treatment and in some cases trended to increase (e.g., IL‐6 and TREM‐2). Although the mechanisms are not clear, it is not uncommon to have expression patterns of both pro‐ and anti‐inflammatory cytokines changing in the same direction following an epileptogenic insult,^26^, ^56^ and there are potential pathways, such as the NfkB pathway,^57^, ^58^ which is known to regulate the expression of both pro‐ and anti‐inflammatory cytokines. Furthermore, studies have shown that canonical proinflammatory cytokines IL‐6 and TREM‐2 can display neuroprotective effects depending on the context of the injury and model.^59^, ^60^, ^61^ Similarly, Tgfb can also be involved in both pro‐ and anti‐inflammatory mechanisms and may contribute to seizure induction. So, the relationship between the effects of CQ on the neuroinflammatory patterns and its antiseizure mechanisms remains complex.

Repurposing CQ as an add‐on ASM appears to be a valid option, as this haloquinoline drug has been used for many years to treat traveler's diarrhea and fungal and protozoal gastrointestinal tract infections.^19^ We acknowledge that a larger double‐blind placebo‐controlled trial will provide a definite answer on the antiseizure efficacy of CQ. Nevertheless, the three adolescents included in our open pilot proof‐of‐concept study suffered from very refractory epilepsy, and a decrease in seizure frequency and severity is clinically meaningful. This was also reflected in the secondary outcomes; a positive impact on quality of life and less severe seizures were seen in all three patients. The efficacy might also have been underestimated, as our data show that CQ plasma levels were low (1–3.5 μg/mL) in all patients.

Predicting effective brain concentrations of unbound (pharmacologically active) CQ based on plasma levels remains challenging due to limited and inconsistent preclinical and clinical pharmacokinetic data. Studies on CQ plasma levels in preclinical models have mainly been conducted in rats. In rats, 100 mg/kg ip yields a mean CQ plasma concentration of 30 nmol/mL (≈9 μg/mL) at .5–1 h,^62^ whereas 200 mg/kg ip results in 58 nmol/mL, indicating dose‐proportional exposure. However, CQ doses of >200 mg/kg ip deviate from dose proportionality. Based on these data, a 10 mg/kg ip dose, the effective dose used in mice in our study, would be expected to produce a peak plasma concentration in the order of 3 nmol/mL (.9 μg/mL), consistent with the observed plasma levels in the DRE patients. CQ is known to bind extensively to plasma proteins, particularly albumin and lipoproteins. The free (unbound) fraction is typically <5%; estimates range from .5% to 2% at therapeutic plasma concentrations. Findings from Alzheimer disease studies suggest that plasma CQ of 4–8 μg/mL are predicted to generate 100–200 nmol·L^−1^ of “active” (free) compound in the brain.^45^ Whether increasing CQ doses in DRE patients would enhance the antiseizure efficacy warrants further investigation.

Although CQ has been used worldwide, there was an outbreak of SMON in the 1950s‐1970s in which the majority of cases were in Japan, prompting speculation that the unique genetic background of the Japanese population may have contributed to the development of SMON.^40^, ^63^ Specific single nucleotide polymorphisms in the antioxidant protein NQO1 or transporters ABCC4 and ABCC11, mainly found in the Japanese population, were proposed as risk factors for developing SMON.^19^, ^64^ As a consequence, we did not include patients of Asian origin in our study. However, Matsumoto et al.^65^, ^66^ analyzed the potential association between SMON and polymorphisms in *NQO1*, *ABCC4*, or *ABCC11* in 125 SMON patients. They found, however, that the frequencies of the loss‐of‐function of *NQO1*, *ABCC4*, or *ABCC11* alleles in SMON patients and the normal control group did not differ significantly in a multifaceted analysis. The identification of genetic factors associated with SMON development upon CQ overuse will be very important, not only to understand the pathogenesis of SMON, but also for patient stratification in CQ repurposing trials.

## AUTHOR CONTRIBUTIONS

Karin Thevissen conceptualized the study. Jana Tits, Karin Thevissen, Daniëlle Copmans, Annelii Ny, Lieven Lagae, Peter de Witte, Jo Sourbron, Arnout Voet, Peravina Thergarajan, Nigel C. Jones, Idrish Ali, Christine Germeys, Sebastian Perrone, and Virginia Minniti designed the study and methodologies. All authors contributed to figures and manuscript writing.

## CONFLICT OF INTEREST STATEMENT

M.M. has served on the advisory board for Merck and has received speaker honoraria from Merck and Biogen. Her institution receives funding from Merck, Australian National Health Medical Research Council, Brain Foundation, Charles and Sylvia Viertel Foundation, and MS Research Australia. L.V.D.B. is head of the scientific advisory board of Augustine Therapeutics (Leuven, Belgium) and part of the investment advisory board of Droia Ventures (Meise, Belgium). L.L. has received grants as well as speaker/consultant honoraria from Zogenix (now part of UCB Pharma), LivaNova, UCB Pharma, Shire, Eisai, Novartis, and Takeda/Ovid. All other authors declare that they have no competing interests. We confirm that we have read the Journal's position on issues involved in ethical publication and affirm that this report is consistent with those guidelines.

## Supporting information


**FIGURE S1.** Trial design. CQ, clioquinol.
**FIGURE S2.** Nicotinamide adenine dinucleotide (NADH) generated by phosphoglycerate dehydrogenase upon incubation with 20–200 μmol·L^−1^ clioquinol (CQ) in the presence of deproteinized cell extract. NADH was measured spectrophotometrically. Data shown are mean ± SD (*n* ≥ 5 in two biological repeats). Statistical differences: **p < .01 by Kruskal–Wallis test followed by Dunn multiple comparisons test.
**FIGURE S3.** Concentration dependency of truncated serine phosphoglycerate dehydrogenase (sPHGDH) activation by clioquinol (CQ). The catalytic activity of sPHGDH in the presence of 0–5 mmol·L^−1^ 3‐phosphoglycerate (3‐PG) was investigated by following the nicotinamide adenine dinucleotide‐induced colorimetric change of resazurin, using the experimental setup as in Figure 1C. The initial rate was calculated in the first 15 min at the linear range of the reaction in three independent experiments and is indicated as mean ± SD.
**FIGURE S4.** Intracellular glycine and glutamate levels in astrocytes. Intracellular glycine and glutamate abundances are shown per microgram protein ± SD in induced pluripotent stem cell‐derived astrocytes treated with vehicle (0 nmol·L^−1^, *n* = 6), 62.5 nmol·L^−1^ clioquinol (CQ; *n* = 3), or 125 nmol·L^−1^ CQ (*n* = 3) for 50 h in three separate astrocyte differentiations. Statistical differences: **p < .01 by one‐way analysis of variance with Dunnett multiple comparisons test.
**FIGURE S5.** Phosphoglycerate dehydrogenase inhibitor CBR‐5884 induces epileptiform brain activity in 7 days postfertilization wild‐type zebrafish larvae. Electrophysiological seizure activity (10 min noninvasive local field potential recording) is expressed in number of epileptiform events ± SD; polyspiking events (≥three spikes) with ≥three times the amplitude of the baseline and lasting ≥50 ms. Incubation time was 45 min, based on the time‐to‐peak locomotor effect (data not shown). Numbers of recordings analyzed were vehicle (*n =* 10), 1 μmol·L^−1^ CBR‐5884 (*n* = 10). Statistical differences: ***p < .001 by Mann–Whitney test.
**FIGURE S6.** Representative local field potential recordings of 7 days postfertilization zebrafish. Zebrafish were treated with 1% dimethylsulfoxide (vehicle [VHC]) or .25–1 μmol·L^−1^ clioquinol (CQ), in the absence or presence of .64 μmol·L^−1^ phosphoglycerate dehydrogenase inhibitor CBR‐5884, followed by the addition of ethyl ketopentenoate (EKP).
**FIGURE S7.** Glycine and serine levels in zebrafish heads of (A, D) wild‐type and (B, E) ethyl ketopentenoate (EKP)‐treated larvae, and in (C, F) Dravet syndrome zebrafish, with or without 1 μmol·L^−1^ clioquinol (CQ). Results are expressed as normalized glycine amount per 10 homogenized heads ± SD. Statistical differences are by unpaired Student *t*‐test (A, B, E), one‐way analysis of variance with Tukey multiple comparisons test (C, F), and Mann–Whitney test (D). ns, not significant.
**FIGURE S8.** Antiseizure activity analysis of fenfluramine (FA) in the mouse 6‐Hz (44 mA) psychomotor seizure model. Drug‐resistant psychomotor seizures were induced by electrical stimulation through the cornea, 60 min after intraperitoneal injection of vehicle (*n* = 9), FA (5 mg/kg, *n* = 6), and FA (20 mg/kg, *n* = 6). Mean seizure durations (± SD) are depicted. Statistical differences: **p < .01 and ***p < .001 and by one‐way analysis of variance with Dunnett multiple comparisons test.
**TABLE S1.** Effect of clioquinol (CQ) on proinflammatory genes.

## Data Availability

The data that support the findings of this study are available from the corresponding author upon request.
