# Supplementary material for: Clioquinol as a new therapy in epilepsy: From preclinical evidence to a proof‐of‐concept clinical study
Source: Epilepsia. 2025 Jul 5;66(10):3769–84. doi: 10.1111/epi.18536 (PMC12605802; doi:10.1111/epi.18536)
Supplement: Supplementary file 1 — FIGURE S1. Trial design. CQ, clioquinol. FIGURE S2. Nicotinamide adenine dinucleotide (NADH) generated by phosphoglycerate dehydrogenase upon incubation with 20–200 μmol·L−1 clioquinol (CQ) in the presence of deproteinized cell extract. NADH was measured spectrophotometrically. Data shown are mean ± SD (n ≥ 5 in two biological repeats). Statistical differences: **p < .01 by Kruskal–Wallis test followed by Dunn multiple comparisons test. FIGURE S3. Concentration dependency of truncated serine phosphoglycerate dehydrogenase (sPHGDH) activation by clioquinol (CQ). The catalytic activity of sPHGDH in the presence of 0–5 mmol·L−1 3‐phosphoglycerate (3‐PG) was investigated by following the nicotinamide adenine dinucleotide‐induced colorimetric change of resazurin, using the experimental setup as in Figure 1C. The initial rate was calculated in the first 15 min at the linear range of the reaction in three independent experiments and is indicated as mean ± SD. FIGURE S4. Intracellular glycine and glutamate levels in astrocytes. Intracellular glycine and glutamate abundances are shown per microgram protein ± SD in induced pluripotent stem cell‐derived astrocytes treated with vehicle (0 nmol·L−1, n = 6), 62.5 nmol·L−1 clioquinol (CQ; n = 3), or 125 nmol·L−1 CQ (n = 3) for 50 h in three separate astrocyte differentiations. Statistical differences: **p < .01 by one‐way analysis of variance with Dunnett multiple comparisons test. FIGURE S5. Phosphoglycerate dehydrogenase inhibitor CBR‐5884 induces epileptiform brain activity in 7 days postfertilization wild‐type zebrafish larvae. Electrophysiological seizure activity (10 min noninvasive local field potential recording) is expressed in number of epileptiform events ± SD; polyspiking events (≥three spikes) with ≥three times the amplitude of the baseline and lasting ≥50 ms. Incubation time was 45 min, based on the time‐to‐peak locomotor effect (data not shown). Numbers of recordings analyzed were vehicle (n = 10), 1 μmol·L−1 CBR‐58 [file EPI-66-3769-s001.docx]

Clioquinol as a new therapy in epilepsy: from preclinical evidence to a proof-of-concept clinical study

**Supplementary materials**

**Materials and Methods**

**PHGDH enzymatic assay in cellular context based on direct NADH readout**

Recombinant human PHGDH and PSAT1 enzymes with N-terminal His_6_ tags were expressed in *E. coli* BL21(DE3) cells and purified as described,(49) except for an additional gel filtration step using a Superdex 200 10/30 GL column (GE Healthcare) at a 0.75 ml/min flow rate in 25 mM Tris (pH 7.5) and 150 mM NaCl. Protein concentration was determined via A_280_ and using an extinction coefficient estimated with ProtParam (RRID: SCR_018087). Protein purity was estimated at > 95% based on SDS-PAGE. Purified proteins were stored at -80°C with 10% glycerol.

HAP1 cells (Horizon Discovery Group, Austria) were cultured in IMDM with penicillin (100 units/ml), streptomycin (100 µg/ml), and 10% FBS (Gibco) at 37°C and 5% CO_2_. Forty-eight hours after seeding (1.5 x 10^6^ per 10 cm dish with 10 ml IMDM), cells were washed with PBS (at 37°C) and lysed by scraping into 400 µl of ice-cold 50 mM HEPES (pH 7.1). Lysates underwent three freeze-thaw cycles, centrifugation (30 min at 17,000 x *g* and 4°C), and heat treatment at 95°C (5 min) for protein denaturation, followed by centrifugation to yield “deproteinized cell extracts” that were used on the same day.

PHGDH activity was measured spectrophotometrically at 340 nm (NADH formation; e = 6220 M^-1^ cm^-1^) using a microplate reader. Reactions (200 µl final volume) contained 25 mM Tris (pH 9), 1 mM dithiothreitol, 1 mM MgCl_2_, 5 µM pyridoxal phosphate, 1 mM L-glutamate, 400 mM KCl, 0.5 mM NAD^+^, 100 µg/ml PSAT1, 10 µg/ml PHGDH, 100 µl of deproteinized HAP1 cell extract, 20 µM or 200 µM CQ (or 2% DMSO), and 300 µM 3-phosphoglycerate. The mixture was incubated at 37°C for 10 minutes in the absence of 3-phosphoglycerate before initiating the reaction by substrate addition to determine initial velocities. Kruskal-Wallis test followed by Dunn's multiple comparisons test was used to determine statistical significance.

**Determination of glycine and glutamate levels in iPSC-derived astrocytes**

Control induced pluripotent stem cell (iPSCs) were generated by genetic correction of an iPSC line derived from a 17-year-old male amyotrophic lateral sclerosis (ALS) patient using CRISPR-Cas9.(50) iPSCs were cultured on Geltrex (Gibco, A1413301) in Complete Essential 8 medium (Gibco, A1517001) with 1% penicillin/streptomycin (Gibco, 15070063). iPSCs were differentiated in mature astrocytes as described.(51) After 28 days (d+28) of maturation, mature astrocytes were plated in Geltrex-coated 12-well plates at 300.000 cells per well for glutamate and glycine determination.

After 30 days of maturation (d+30), mature astrocytes were treated with 125 nM CQ, 62.5 nM CQ or 1% DMSO for 50h in AMM without FBS containing 5 mM glucose (Sigma-Aldrich, G7021) and 2mM lactate (Sigma-Aldrich, 71718). After washing with ice cold 0.9% NaCl solution, metabolites were extracted using 300 μL of an 80% methanol extraction buffer containing 2 μM of deuterated (d27) myristic acid as internal standard. Protein concentrations were determined using the micro-BCA kit (23235, Pierce Biotechnology, Rockford, US). Following extraction, samples were centrifuged at 20.000×g for 20 min at 4 °C. Glutamate and glycine abundances were determined in the supernatant using mass spectrometry. Briefly, 10 µl of supernatant was loaded into a Dionex UltiMate 3000 LC System (Thermo Scientific Bremen, Germany) equipped with a C-18 column (Acquity UPLC -HSS T3 1. 8 µm; 2.1 x 150 mm, Waters) coupled to a Q Exactive Orbitrap mass spectrometer (Thermo Scientific) operating in negative ion mode. A step gradient was carried out using solvent A (‘A’, 10 mM TBA and 15 mM acetic acid) and solvent B (‘B’, 100% methanol), starting at 5% B and 95% A until 2 min post injection. A linear gradient to 37% B was carried out until 7 min, increasing to 41% until 14 min, and further to 95% B until 26 min. At 30 min the gradient returned to 5% B. The flow was kept constant at 0.25 mL/min and the column at 40°C throughout the analysis. The MS operated in full scan mode (m/z range: [70.0000-1050.0000]) using a spray voltage of 4.80 kV, capillary temperature of 300 °C, sheath gas at 40.0, auxiliary gas at 10.0. The AGC target was set at 3.0E+006 using a resolution of 140000, with a maximum IT fill time of 512 ms. Data collection was performed using the Xcalibur software (Thermo Scientific). The data analyses were performed by integrating the peak areas (El-Maven – Polly - Elucidata) and normalized to sample protein concentration. One-way ANOVA with Dunnett’s multiple comparisons test was performed to assign significant differences.

**Figures and Tables**

**
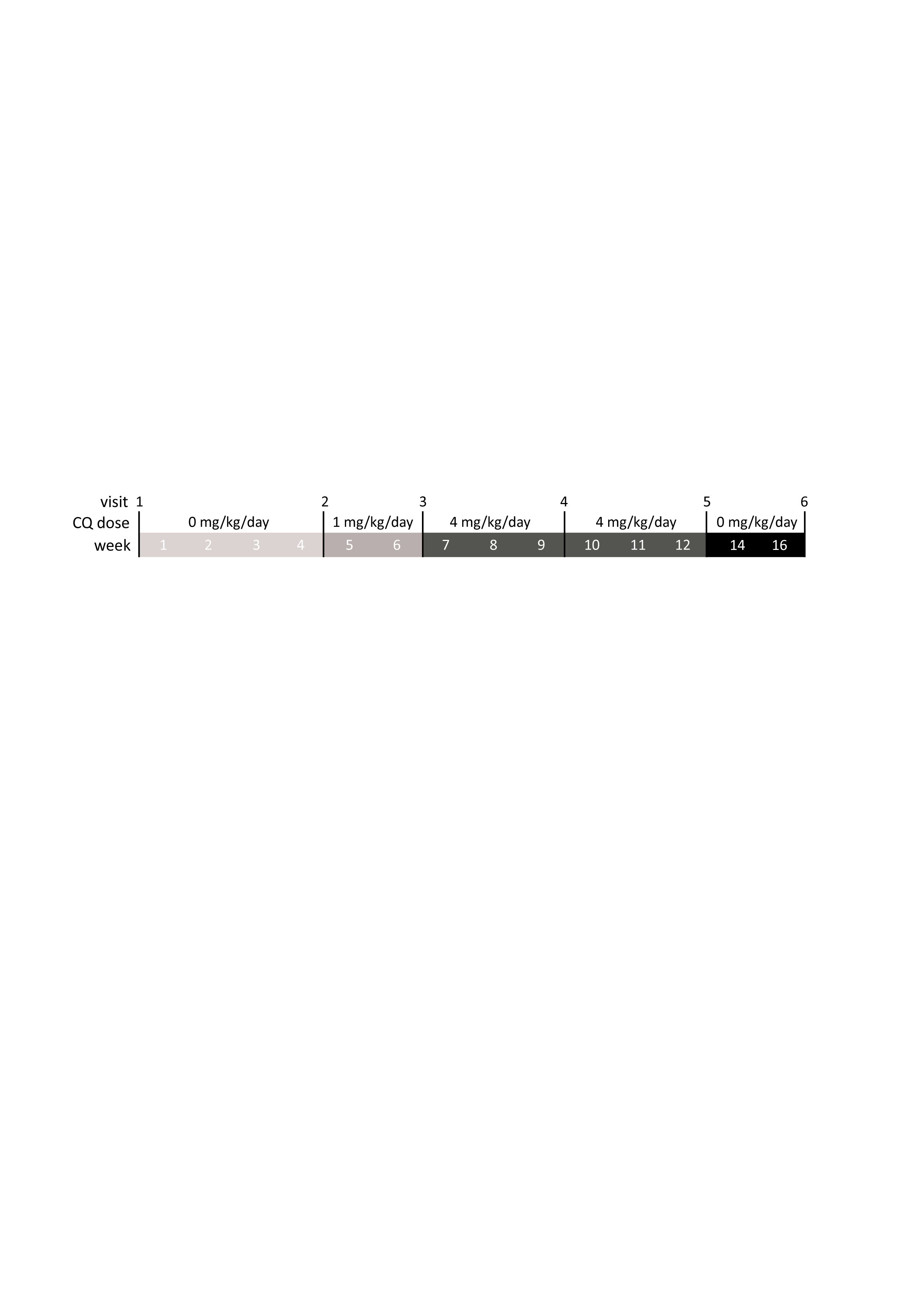
**

**Supplementary Figure 1. Trial Design**

**
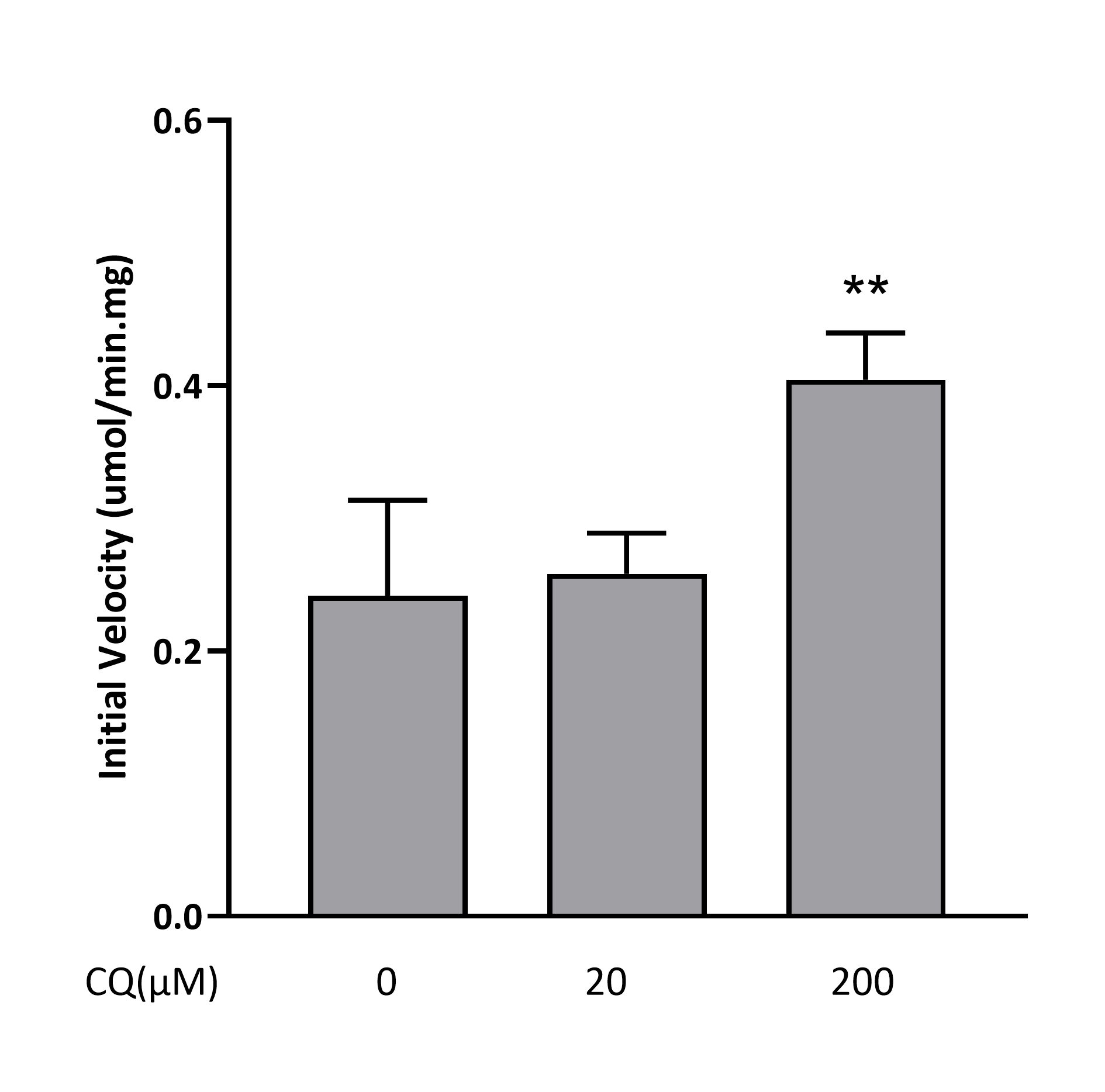
**

**Supplementary Figure 2.** NADH generated by PHGDH upon incubation with 20-200 µM CQ in the presence of deproteinized cell extract. NADH was measured spectrophotometrically. Data shown are means ± SD (*n* ≥ 5 in 2 biological repeats). Statistical differences: ***P*<0.01 by Kruskal-Wallis test followed by Dunn's multiple comparisons test.

**
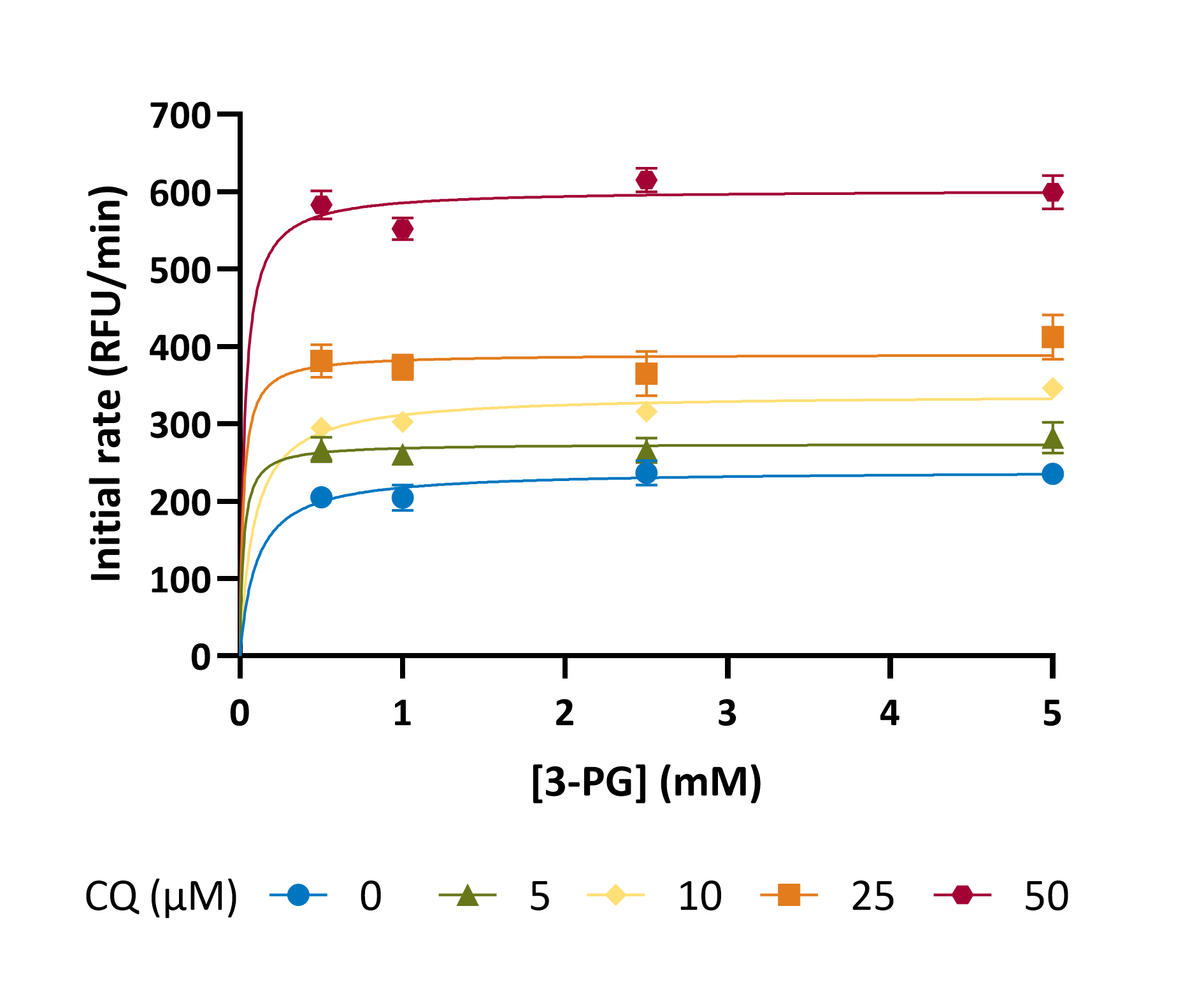
**

**Supplementary Figure 3. Concentration dependency of sPHGDH activation by CQ.** The catalytic activity of sPHGDH in the presence of 0-5 mM 3-phosphoglycerate (3-PG) was investigated by following the NADH-induced colorimetric change of resazurin; using the experimental setup as in Fig. 1C. The initial rate was calculated in the first 15 minutes at the linear range of the reaction in three independent experiments and is indicated as mean ± SD.


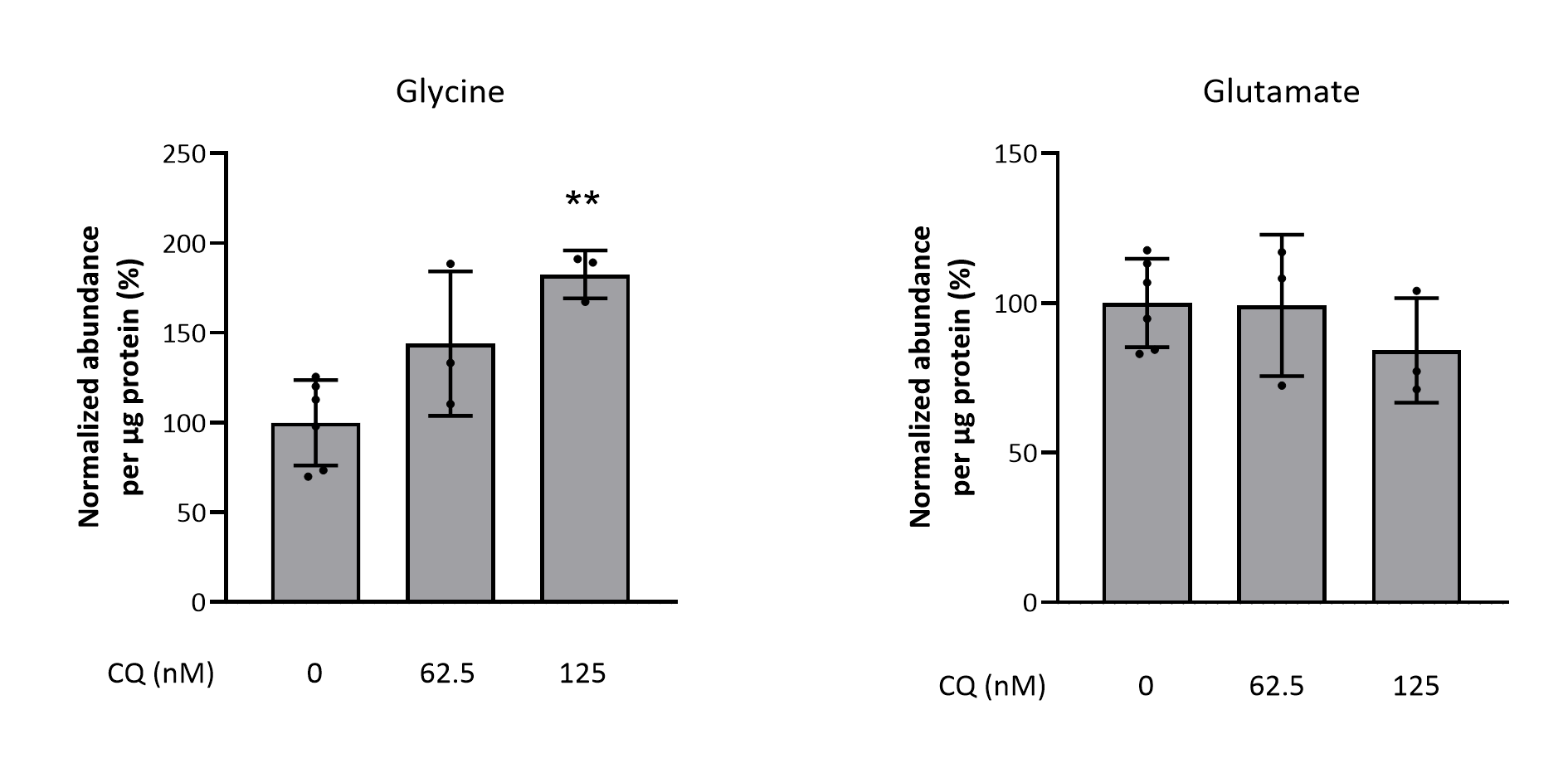


**Supplementary Figure 4. Intracellular glycine and glutamate levels in astrocytes.** Intracellular glycine and glutamate abundances per µg protein ± SD in iPSC-derived astrocytes treated with vehicle (0 nM, *n* = 6), 62.5 nM (*n* = 3) or 125 nM CQ (*n* = 3) for 50 h in 3 separate astrocyte differentiations. Statistical differences: ***P*<0.01 by one-way ANOVA with Dunnett’s multiple comparisons test.


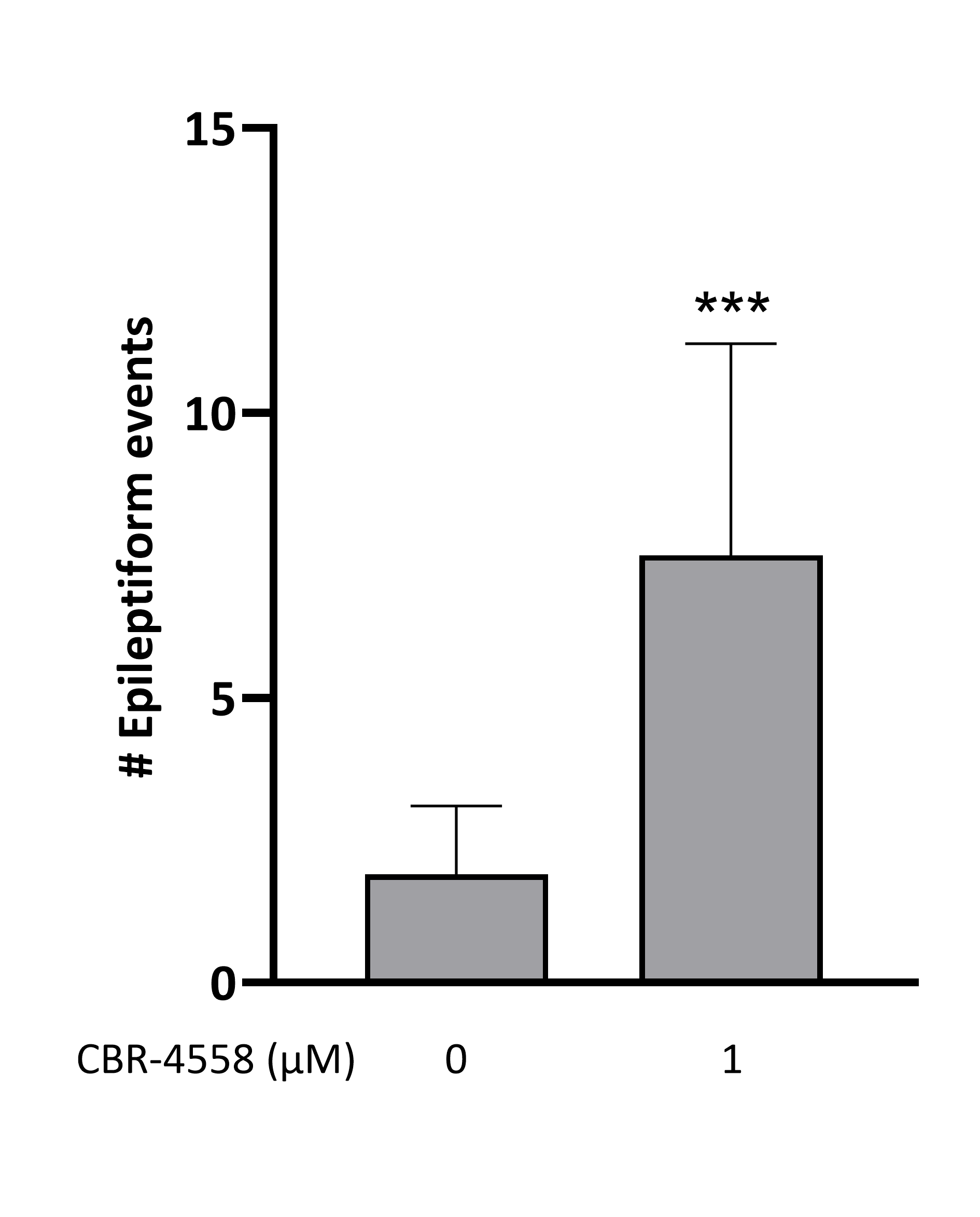


**Supplementary Figure 5. PHGDH inhibitor CBR-5884 induces epileptiform brain activity in seven dpf wild-type zebrafish larvae.** Electrophysiological seizure activity (10 min non-invasive local field potential recording) is expressed in number of epileptiform events ±SD; polyspiking events (≥ three spikes) with ≥ three times the amplitude of the baseline and lasting ≥ 50 ms. Incubation time was 45 minutes, based on the time-to-peak locomotor effect (data not shown). Number of recordings analyzed were VHC (*n*  = 10), 1 µM CBR-5884 (*n* = 10). Statistical differences: ****P*<0.001 by Mann Whitney test.


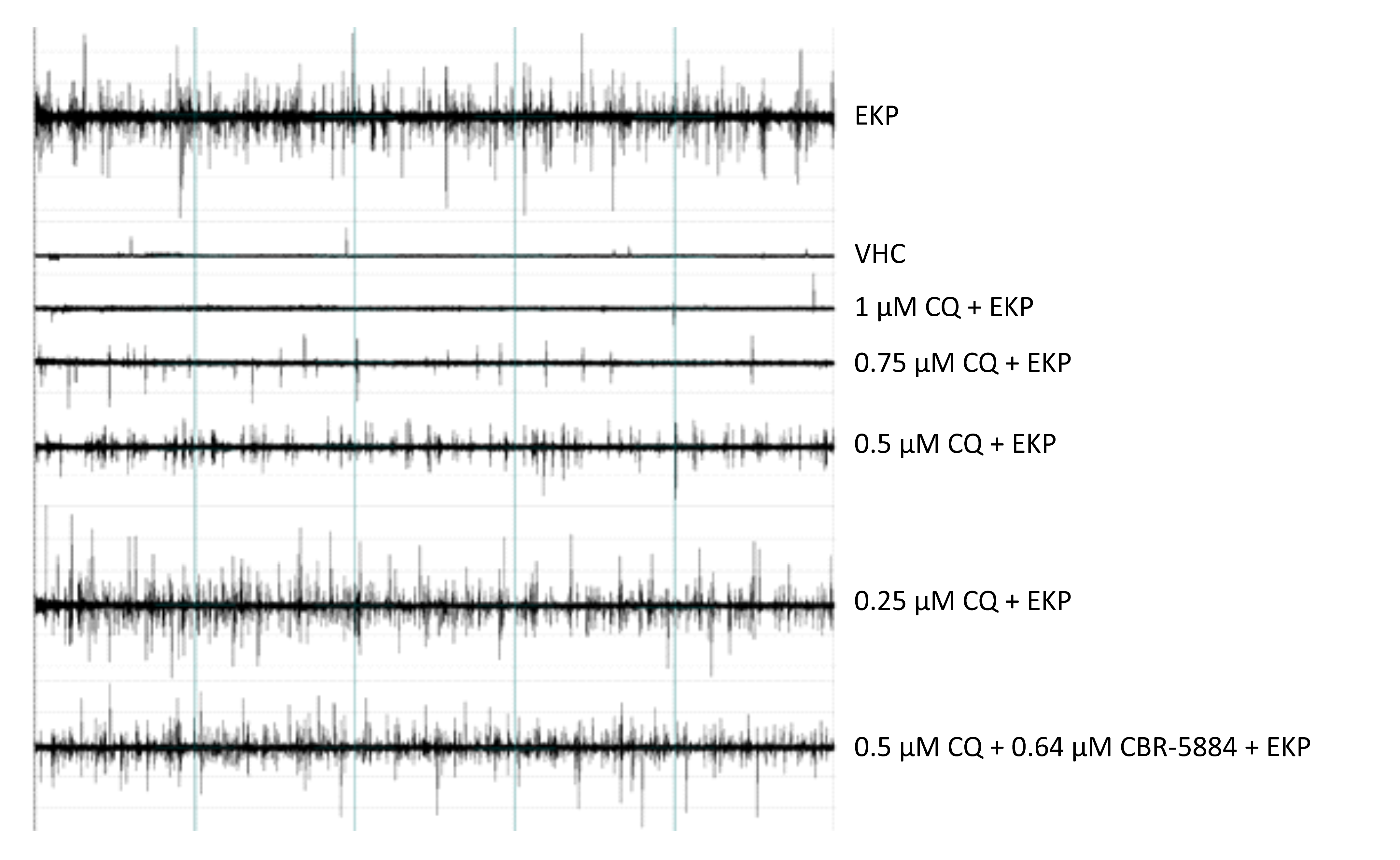


**Supplementary Figure 6. Representative LFP recordings of seven dpf zebrafish**. Zebrafish were treated with 1% DMSO (VHC) or 0.25 – 1 µM clioquinol, in the absence or presence of 0.64 µM Phgdh inhibitor CBR-5884, followed by the addition of EKP.


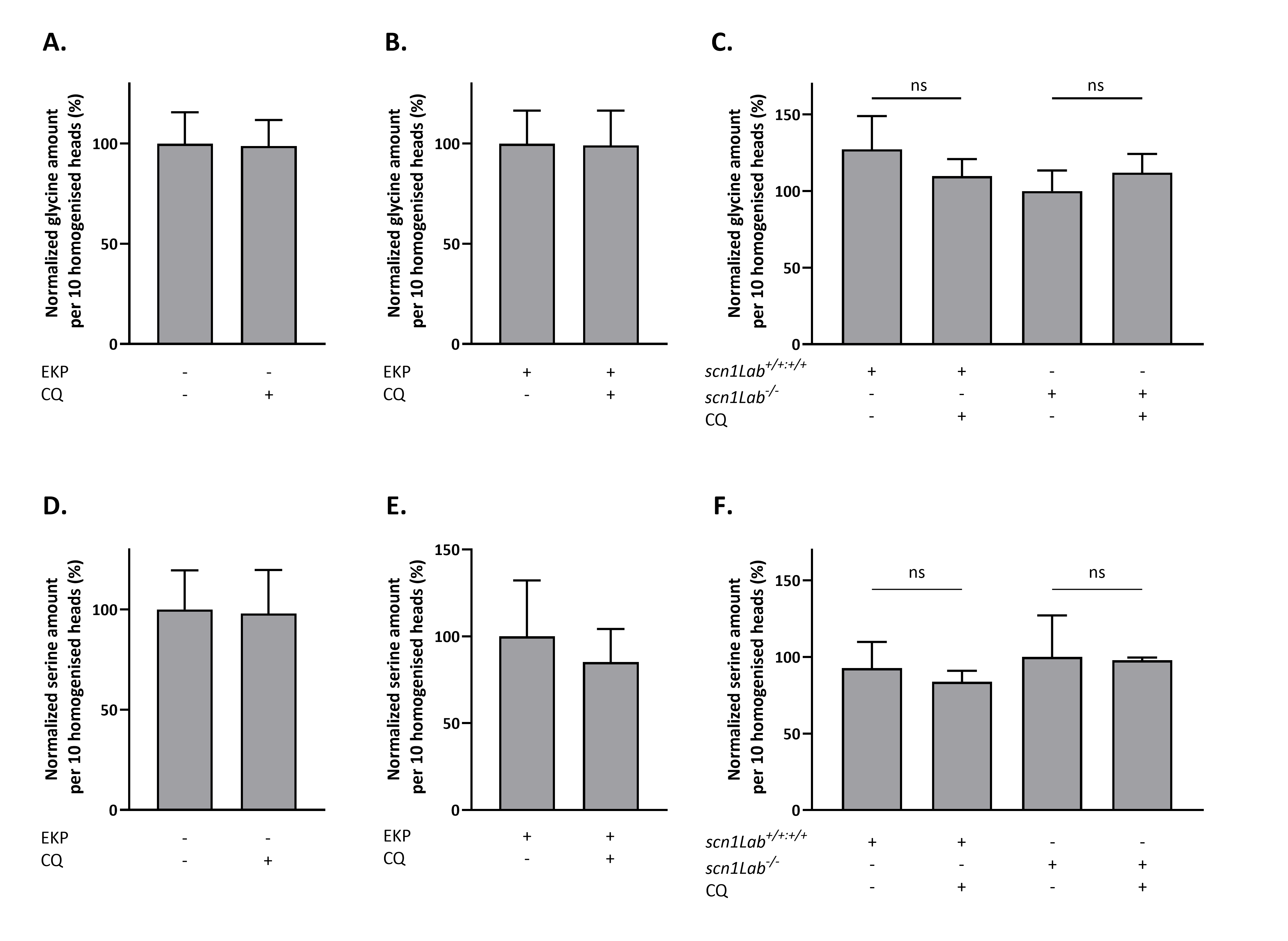


**Supplementary Figure 7. Glycine and Serine levels in zebrafish heads**. Glycine and Serine levels in heads of (**A, D**) wild-type and (**B, E**) EKP treated larvae, and in (**C, F**) Dravet syndrome zebrafish, with or without 1 µM CQ. Results expressed as normalized glycine amount per ten homogenised heads ± SD. Statistical differences by unpaired Student’s t-test (**A, B, E)**, one-way ANOVA with Tukey’s multiple comparisons test (**C, F**) and Mann Whitney test (**D**).


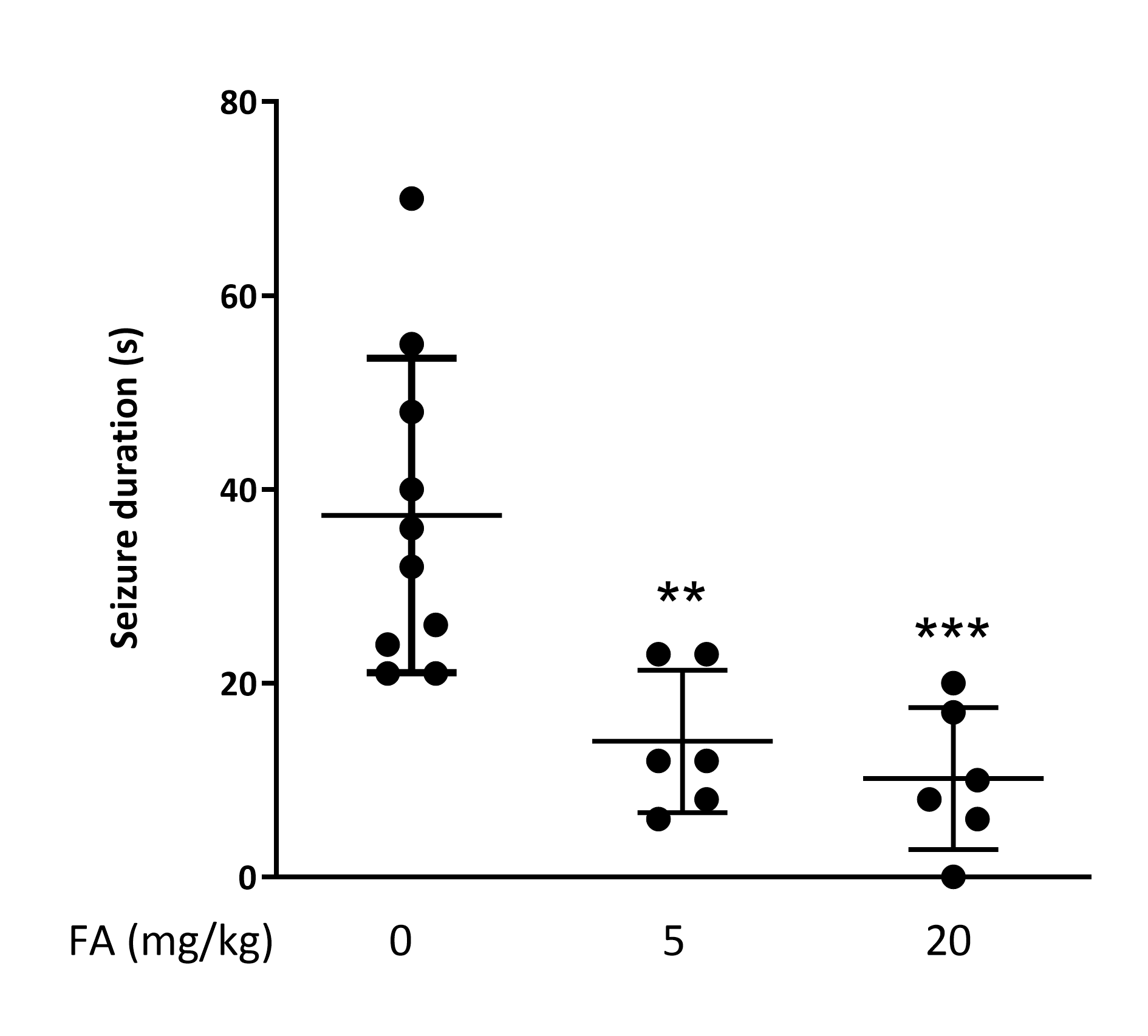


**Supplementary Figure 8. Antiseizure activity analysis of fenfluramine (FA) in the mouse 6-Hz (44 mA) psychomotor seizure model.** Drug-resistant psychomotor seizures were induced by electrical stimulation through the cornea, 60 min after i.p. injection of vehicle (VHC, *n* = 9), FA (20 mg/kg, *n* = 6) and FA (20 mg/kg, *n* = 6). Mean seizure durations (± SD) are depicted. Statistical differences: ****P*<0.001 and ***P*<0.01 by one-way ANOVA with Dunnett’s multiple comparisons test.

**Supplementary Table 1. Effect of CQ on pro-inflammatory genes**.

| **Gene** | **Effect of SSSE** | **Effect of CQ treatment** |
| --- | --- | --- |
| Il1β | Upregulated  (*P* = 0.0003) | No change (*P* = 0.7) |
| Il1α | Upregulated  (*P* = 0.0002) | No change (*P* = 0.13) |
| Tnfα | Upregulated  (*P* = 0.003) | No change (*P* = 0.25) |
| Cd86 | Upregulated  (*P* = 0.0001) | No change (*P* = 0.2) |
| IL6 | Upregulated  (*P* = 0.009) | Trend to increase (*P* = 0.06) |
| Trem2 | Upregulated  (*P* = 0.0001) | Trend to increase (*P* = 0.06) |
